# Racismos y salud mental en jóvenes indígenas residentes en la Zona Metropolitana de Oaxaca, México

**DOI:** 10.18294/sc.2024.4908

**Published:** 2024-12-02

**Authors:** María Alejandra Sánchez Bandala, Paola María Sesia

**Affiliations:** 1 Doctora en Antropología. Profesora-investigadora, Universidad de la Sierra Sur; Miahuatlán de Porfirio Díaz, México. alejandra.bandala1@gmail.com Universidad de la Sierra Sur Universidad de la Sierra Sur Miahuatlán de Porfirio Díaz Mexico alejandra.bandala1@gmail.com; 2 Doctora en Antropología. Profesora-Investigadora, Centro de Investigaciones y Estudios Superiores en Antropología Social-Pacífico Sur; Oaxaca de Juárez, México. sesia@ciesas.edu.mx Centro de Investigaciones y Estudios Superiores en Antropología Social-Pacífico Sur Centro de Investigaciones y Estudios Superiores en Antropología Social-Pacífico Sur Oaxaca de Juárez Mexico sesia@ciesas.edu.mx

**Keywords:** Pueblos Indígenas, Salud Mental en Grupos Étnicos, Servicios de Salud Mental, Racismo, México

## Abstract

Las poblaciones indígenas presentan altas prevalencias de trastornos mentales y limitado acceso a servicios de salud mental. El objetivo del estudio fue analizar las trayectorias de atención a trastornos mentales de jóvenes indígenas residentes en la Zona Metropolitana de Oaxaca, México. Entre mayo y agosto de 2023, se llevó a cabo un estudio cualitativo basado en observación no participante, entrevistas en profundidad a siete personas jóvenes indígenas y entrevistas semiestructuradas a nueve personas profesionales de la salud, curanderas o responsables de grupos de ayuda mutua. Se identificaron procesos de occidentalización, no exentos de tensiones, en la forma de concebirse como jóvenes e indígenas, en el desarrollo de sus trastornos mentales y en la atención de estos, para lo cual utilizaron servicios psicológicos, psiquiátricos, grupos de ayuda mutua y, de manera limitada, medicina tradicional. Se concluye que en estos procesos se articulan y potencian racismos interpersonales, institucionales y epistémicos, que será necesario desarticular para mejorar la salud mental de personas jóvenes indígenas.

## INTRODUCCIÓN

La carga creciente de trastornos mentales, aunada a las brechas de tratamiento, constituyen uno de los problemas centrales de la salud pública en la región de las Américas^1^. A pesar de existir un consenso sobre la urgencia de la problemática, no existe una forma unívoca de concebir la salud y la enfermedad mental. Algunas concepciones retoman el modelo médico y privilegian un enfoque biologicista e individual, centrado en la psicopatología^2^. Superando este reduccionismo (aunque no un enfoque individual), la Organización Mundial de la Salud (OMS) define la salud mental como “un estado de bienestar en el que la persona realiza sus capacidades y es capaz de hacer frente al estrés normal de la vida, de trabajar de forma productiva y de contribuir a su comunidad”^3^. Por su parte, desde la salud colectiva latinoamericana se concibe la enfermedad -incluyendo la mental- como un marcador múltiple que remite a los órdenes biológico, cultural, histórico y social y que se orienta a comprender la “trama colectiva en que se generan y resuelven […] las enfermedades”, abriendo la posibilidad de desarrollar una “terapéutica comprensiva”^4^. De esta manera, la salud mental colectiva se centra en el estudio de las “experiencias de aflicción”, recuperando el “mundo social” del sufrimiento psíquico^5^, atendiendo a las determinaciones sociales de la salud mental, centralmente las violencias impuestas por las condiciones materiales de existencia y las relaciones de dominación; al mismo tiempo que reconoce la capacidad de agencia de las personas con sufrimiento psíquico para la comprensión de sus procesos y su participación en sus cuidados^6^. 

Como puede apreciarse, podemos encontrar diversas formas de conceptualizar la salud y la enfermedad mental, encontrando incluso concepciones antagónicas, por ejemplo, entender a la salud mental como la capacidad -generalmente individual- para adaptarse, afrontar el estrés “normal” de la vida y las adversidades y conservar la armonía en las relaciones^3^ y, desde otras posiciones, entenderla como la capacidad para transformar las condiciones de vida^7^.

Esta polisemia se identifica también en las formas de referirse a aquello que podríamos considerar contrario a la salud mental. El concepto de *enfermedad mental* suele referir a entidades discretas propias de los enfoques biomédicos; sin embargo, este es un concepto cada vez menos utilizado en la literatura académica prefiriéndose el de *trastorno mental* el cual, siguiendo a Braunstein, sigue siendo un eufemismo para referirse a un constructo muy similar al de enfermedad mental^8^. Por su parte, la OMS señala que un trastorno mental “se caracteriza por una alteración clínicamente significativa de la cognición, la regulación de las emociones o el comportamiento de un individuo”^9^. Así, en la definición de un trastorno se suelen incluir criterios de desviación, de limitaciones para la funcionalidad, una experiencia de malestar y cierta idea de peligrosidad (aunque se ha discutido que los dos últimos criterios pueden ser cuestionables)^10^. En términos prácticos, los trastornos mentales siguen haciendo referencia a aquellos constructos que se pueden hacer corresponder a los criterios diagnósticos propuestos en las clasificaciones nosográficas dominantes, como la Clasificación internacional de enfermedades (CIE) y el Diagnostic and Statistical Manual of Mental Disorders (DSM)^11^. 

Desde enfoques críticos se utilizan otras categorías para referirse a aquello que se considera distinto de la salud mental, una de ellas es la de *sufrimiento psíquico*, entendida como una experiencia perjudicial que se percibe como subjetiva, pero se origina en la red de relaciones en la que está inmersa la persona^12^. Estos sufrimientos se conciben como parte de los problemas de la vida que, si bien producen desdicha, no se patologizan. Esta categoría tiene cierta afinidad con el concepto de malestar, aunque algunos autores utilizan este último para destacar el conflicto y las contradicciones sociales (no necesariamente registradas de manera consciente por los sujetos) que subyacen a los malestares^13^. En este trabajo se utilizan las categorías de *enfermedad* y *trastorno* para indagar sobre las construcciones que las y los sujetos de estudio conciben en términos de patologías, y las categorías de *sufrimiento* o *malestar* para aquellas experiencias de desdicha no necesariamente patologizadas por los propios sujetos.

Cuando se aborda la salud mental de poblaciones indígenas, es posible identificar dos grandes líneas de pensamiento. Tanto desde la epidemiología, como desde la antropología médica crítica -esta última atenta a las relaciones asimétricas de poder y a las desigualdades estructurales^14^^,^^15^- han identificado que estas poblaciones tienen, a nivel global, altas prevalencias (generalmente mayores que sus contrapartes no indígenas) de trastornos como depresión, consumo de sustancias psicoactivas, alcoholismo y suicidio; condiciones que se han asociado al estrés social derivado de los procesos migratorios^16^, la discriminación, pérdida de elementos culturales y de control sobre sus condiciones de vida^17^. Por ejemplo, un estudio en México acerca de los datos oficiales de mortalidad en 2014, reportó que la tasa nacional de suicidios entre varones indígenas (incluyendo a adolescentes y jóvenes) fue mayor que entre el resto de la población, mientras la tasa de mortalidad relacionada al uso de alcohol fue 2,5 veces más alta entre este grupo en comparación con poblaciones no indígenas^18^. Por otro lado, un estudio en Colombia reportó que la tasa de suicidio era más de cien veces mayor entre la población indígena (500 por 100.000 habitantes) en ese país en comparación con la población colombiana en su conjunto (4,4 por 100.000 habitantes)^19^. También se ha evidenciado que estas poblaciones tienen peores indicadores respecto a la disponibilidad y acceso a servicios de salud biomédicos, debido a su residencia en contextos rurales y sus condiciones de pobreza^20^^,^^21^ y que la atención que reciben en servicios biomédicos es deficiente, pues se desconocen los síndromes de filiación cultural, se tiende a traducir sus malestares a las categorías de la psiquiatría occidental, no se toman en cuenta los aspectos socio-relacionales de estos cuadros nosológicos y se desconocen o rechazan sus recursos terapéuticos comunitarios^19^^,^^22^^,^^23^; a pesar de la existencia en el ámbito internacional y nacional de un amplio marco legal e institucional que promueve un enfoque intercultural y de derechos para la atención a la salud de los pueblos indígenas^24^^,^^25^.

Por su parte, desde abordajes que reconocen la relatividad cultural para la definición y vivencia de la salud y la enfermedad mental, se ha destacado la necesidad de comprender estos procesos como producto de las cosmovisiones de los propios pueblos, y no tratando de hacerlos encajar en los modelos de pensamiento occidentales. Esta línea de trabajo ha evidenciado que en algunos pueblos indígenas no existe un concepto análogo al de “salud mental” y que, en los que existe, su contenido difiere del concepto occidental, centrándose en elementos como la espiritualidad, el buen vivir y/o la armonía con la *comunidad* y la naturaleza^17^. 

Desde disciplinas como la antropología médica, la etnopsiquiatría o la psiquiatría transcultural se ha producido una extensa literatura orientada a la comprensión de “síndromes de filiación cultural” tales como mal de ojo, aire, nervios, susto, envidia, vergüenza, muina o bilis, los cuales se caracterizan por manifestaciones a la vez orgánicas y psíquicas, y se han documentado las diversas prácticas terapéuticas tradicionales^23^^,^^26^^,^^27^^,^^28^^,^^29^. Sin embargo, se ha señalado que algunos de estos estudios centraron su atención solo en la medicina tradicional, sin profundizar en sus articulaciones de facto con la medicina moderna, lo que terminó limitando su capacidad explicativa^30^.

Se plantea entonces la pertinencia de abordar los procesos salud-enfermedad-atención de las poblaciones indígenas asumiendo la importancia del cambio cultural, que se ha agudizado en las últimas décadas debido a procesos como la migración, mayor grado de escolarización y el acceso a diversas tecnologías de la información y la comunicación^31^. Estas condiciones hacen de la población indígena, joven y migrante sujetos privilegiados para explorar los procesos de cambio y su impacto en la manera de concebir la salud y la enfermedad mental y de prevenir y atender las afectaciones en este campo, presumiblemente haciendo uso de diferentes formas de atención. 

Una herramienta teórico-metodológica que resulta útil para abordar la complejidad de los procesos señalados es la categoría *trayectorias de atención*, que puede entenderse como los procesos que llevan a cabo las personas para procurarse una terapia a partir de la aparición de un problema de salud, para lo cual pueden utilizar diferentes instancias terapéuticas^32^. Osorio propone que desde este enfoque se analizan las decisiones y estrategias que despliegan los sujetos para enfrentar -y en ese camino entender- sus episodios de padecer^33^.

La categoría *trayectorias de atención* ha sido utilizada para estudiar diferentes procesos de salud-enfermedad-atención^34^^,^^35^^,^^36^^,^^37^^,^^38^^,^^39^^,^^40^^,^^41^. Estos estudios han puesto de manifiesto el uso secuencial o paralelo de diferentes opciones terapéuticas, la importancia de los procesos de autoatención, y han permitido aproximarse a los significados y experiencias subjetivas de enfermos y familiares. Algunos de estos estudios se han orientado al análisis de los procesos de búsqueda de atención de población indígena o que reside en contextos rurales y han evidenciado el sincretismo y complementariedad de saberes biomédicos y tradicionales^42^^,^^43^^,^^44^^,^^45^. Sin embargo, los estudios sobre *trayectorias de atención* en el campo de la salud mental siguen siendo escasos tanto a nivel internacional^46^^,^^47^^,^^48^^,^^49^ como en México ^50^, y más aun los que se realizan con población indígena^51^. 

El contenido de este artículo se deriva de una investigación mayor titulada “Trayectorias de atención a la salud mental en jóvenes indígenas residentes en la Zona Metropolitana de Oaxaca (ZMO)”. En el estado de Oaxaca la carga epidemiológica de varios trastornos mentales presenta una curva ascendente y hay trastornos que se manifiestan de manera frecuente entre la población juvenil: la incidencia de depresión en mujeres ha aumentado desde el 2014 al 2022 (pasando de una tasa de 47 a una de 77 por 100.000 habitantes)^52^, mientras que los suicidios tienen una mayor incidencia entre los jóvenes de los 15 a los 29 años, sobre todo varones^53^. La entidad federativa cuenta con el mayor porcentaje de población hablante de lengua indígena a nivel nacional (el 31,2% frente al 6,1% que es la media nacional), el 69% de la población se autoadscribe como indígena^54^, y presenta un importante desplazamiento rural-urbano^55^. La Zona Metropolitana de Oaxaca está conformada por Oaxaca de Juárez, ciudad capital con mayor población indígena del estado, y 22 municipios aledaños. En cuanto a la disponibilidad de servicios públicos de atención, el estado cuenta con un único hospital psiquiátrico “Cruz del Sur” perteneciente a la Secretaría de Salud y ubicado en la zona conurbada al sur de la ciudad de Oaxaca, siete Unidades de Especialidades Médicas en Centros de Atención Primaria en Adicciones UNEMA-CAPA, de las cuales dos se encuentran en la Zona Metropolitana de Oaxaca, y dos Centros de Integración Juvenil, de los cuales uno está en la ZMO^56^. Para los derechohabientes del Instituto Mexicano del Seguro Social, este organismo ofrece consultas psicológicas en las tres Unidades de Medicina Familiar de primer nivel ubicadas en la Zona Metropolitana de Oaxaca y consulta psiquiátrica en el Hospital General de Zona ubicado en la ciudad de Oaxaca^57^. La mayor oferta es representada por los grupos de ayuda mutua: en la ciudad de Oaxaca y zona conurbada, se encuentran 81 centros de grupos de Alcohólicos Anónimos^58^ y 18 centros de Neuróticos Anónimos^59^.

Se planteó que este espacio geográfico-sociocultural, al ser un polo de atracción de migrantes internos -principalmente provenientes de zonas rurales de la región- y de migrantes internacionales en tránsito y tener una autoproclamada identidad multicultural, configura un caso de interés para la exploración del objeto de estudio. En este artículo el interés se centró en analizar las maneras en que la condición étnica y la estructura sociocultural en la que se inscribe -o mejor dicho, se construye- configuran maneras particulares de enfermar y atender los padecimientos en el campo de la salud mental, en el marco de las trayectorias analizadas. 

## METODOLOGÍA

Se desarrolló un abordaje cualitativo. Los sujetos de estudio fueron jóvenes indígenas, de entre 18 y 30 años, residentes de la Zona Metropolitana de Oaxaca, que reportaran haber buscado atención para un problema de “salud mental” y profesionales de la salud, curanderas o responsables de grupos de ayuda mutua que se desempeñaban en algunos de los espacios que ofrecen atención a la salud mental en la zona de estudio. Cabe señalar que cuatro de estos actores tenían una relación terapéutica con las y los jóvenes entrevistados. Se realizó también observación no participante en los espacios en los que se solicitó colaboración, para establecer contacto con los actores a entrevistar. El trabajo de campo se desarrolló de principios de mayo hasta finales de agosto de 2023.

Con las y los jóvenes (cuatro mujeres y tres hombres) se realizaron entrevistas en profundidad orientadas a conocer sus trayectorias de atención. Con los profesionales (cinco participantes), curanderas (dos participantes) o responsables de grupos de ayuda mutua (dos participantes) se llevaron a cabo entrevistas semiestructuradas orientadas a conocer los rasgos centrales de la atención que se ofrece a las personas jóvenes (indígenas o en general) que presentan trastornos mentales. En la Tabla 1 y la Tabla 2 se presentan las características de las personas participantes. 

**Tabla 1:** Características generales de las personas participantes (jóvenes indígenas). Zona Metropolitana de Oaxaca, México, 2023.

Pseudónimo	Edad	Estado civil	Lugar de nacimiento	Experiencia migratoria	Lugares de residencia	Lengua indígena	Religión	Nivel de estudios	Ocupación	Derecho-habiencia
Alma	22	Soltera	Ciudad Juárez Chihuahua	A los 16 años migró a la ZMO para escapar del acoso escolar en su localidad de origen	Oaxaca de Juárez (ZMO)	Ninguna	Se asume como católica pero casi no la practica	Estudiante de licenciatura	Estudiante	Ninguna
Julia	29	Casada	Santa Catarina Juquila	A los 15 años migró a la ZMO para estudiar el bachillerato	San Bartolo Coyotepec (ZMO)/Miahuatlán de Porfirio Díaz	Chatino	Católica	Licenciatura en Enfermería	Enfermera	ISSSTE
Paula	28	Unión libre	Asunción Cacalotepec	A los 13 años migró a la ZMO para estudiar la secundaria	San Sebastián Tutla (ZMO)/ Miahuatlán de Porfirio Díaz	Ayuuk (mixe)	Católica	Licenciatura en Enfermería	Estudiante de posgrado	IMSS-Bienestar
Magali	29	Soltera	Cuilapam de Guerrero (ZMO)	Ninguna (nació y vive actualmente en la ZMO)	Cuilapam de Guerrero (ZMO)	Ninguna	Ninguna	Licenciatura en Arqueología	Arqueóloga	Ninguna
Fernando	24	Soltero	San Juan Quiahije, Juquila	A los 12 años migró a la ZMO para estudiar la secundaria	Oaxaca de Juárez (ZMO)	Chatino	Católica	Pasante de Ingeniería	Técnico de mantenimiento de máquinas industriales	IMSS-Bienestar
Ismael	29	Unión libre	Santiago Atitlán	A los 27 años migró a la ZMO en busca de atención en la Casa Hogar de Neuróticos Anónimos	Oaxaca de Juárez (ZMO) / Santiago Atitlán	Mixe	Católica	Bachillerato trunco	Músico	Al parecer IMSS-Bienestar (no estaba seguro)
Nicolás	26	Soltero	Miahuatlán de Porfirio Díaz	Migró a la ZMO a los 5 años, junto con su familia, quienes buscaban mejores oportunidades laborales	Santa María Atzompa (ZMO)	Zapoteco (solo entiende pocas palabras)	Ninguna	Bachillerato y carrera trunca	Mecánico	Ninguna

Fuente: Elaboración propia. ZMO = Zona Metropolitana de Oaxaca. ISSSTE = Instituto de Seguridad y Servicios Sociales de los Trabajadores del Estado. IMSS-Bienestar = Subsistema del Instituto Mexicano del Seguro Social orientado a la atención de la población sin seguridad social.

**Tabla 2:** Características generales de las personas participantes (profesionales, curanderas y responsables de grupos de ayuda mutua). Zona Metropolitana de Oaxaca, México, 2023.

Participante	Género	Edad	Lengua indígena	Formación
Curandera y partera	Mujer	84	Zapoteco	Empírica
Curandera	Mujer	60	Zapoteco	Empírica, diplomada en Herbolaria
Psicóloga práctica privada	Mujer	30	Entiende un poco de mixe, pero no lohabla	Licenciada en Psicología
Psicóloga servicio público 1	Mujer	-	Ninguna	Licenciada en Psicología Clínica,especialista en Pareja y Familia
Psicóloga servicio público 2	Mujer	32	Ninguna	Licenciada en Psicología
Psicólogo servicio público	Hombre	30	Ninguna	Licenciado en Psicología, maestrante en Psicoterapia Humanista
Psiquiatra práctica privada	Mujer	30	Entiende muy poco zapoteco	Médico cirujano, especialista enPsiquiatría
Representante de grupo de AlcohólicosAnónimos	Hombre	48	Entiende un poco de zapoteco	Licenciatura (no relacionada con Salud)
Representante de grupo de NeuróticosAnónimos*	Mujer	37	Mazateco	Licenciatura (no relacionada con Salud)

Fuente: Elaboración propia. * Se construyeron códigos similares a los últimos ocho para aplicarlos a cada una de las formas de atención a las que recurrieron las y los sujetos de estudio: medicina tradicional, atención biomédica, atención psicológica, atención psiquiátrica y grupos de ayuda mutua.

Para el desarrollo de las guías de entrevista y el plan de análisis se llevó a cabo un proceso inicialmente deductivo, en el cual se partió de un conjunto de temas a indagar derivados de los objetivos y el estado del arte, y se mantuvo la apertura para la inclusión de líneas temáticas emergentes. Las líneas temáticas *a priori* estuvieron relacionadas con los criterios para identificar(se) como joven indígena, las concepciones sobre la salud y la enfermedad/trastorno mental, las condiciones de vida y su impacto en la salud-enfermedad/trastorno mental, representaciones sobre el trastorno asumido, y las trayectorias de atención. A partir de estas líneas -que se asumieron como las categorías para desarrollar el análisis- se creó un conjunto de códigos. La presentación de los resultados se estructuró de acuerdo con las líneas temáticas señaladas. La Tabla 3 describe la correspondencia entre los subtítulos, las líneas temáticas y los códigos. Aquellos códigos que se muestran en cursiva emergieron del análisis de los datos y se integraron a los códigos siguiendo un procedimiento inductivo. Más que temas no previstos de manera *a priori*, se trató de formas no esperadas de conceptualizar los procesos de interés para el estudio. Por ejemplo, inicialmente se pensaba indagar el impacto de las condiciones de vida en la salud-enfermedad/trastorno mental; sin embargo, se evidenció la necesidad de crear un código que permitiera además apuntar a aquellas experiencias de desdicha no consideradas patológicas por los participantes, por lo que se prefirió utilizar el concepto de *condicionantes de sufrimiento psíquico* para nombrar a dicho código. De igual manera, se consideraba manejar un código que refiriera al diagnóstico de los sujetos participantes, pero dado que muchos de ellos en realidad habían realizado un autodiagnóstico (no elaborado por un profesional) se modificó dicho código y se nombró como *diagnóstico asumido y manifestaciones*. Cabe puntualizar que estas modificaciones no son solo a nivel de los nombres de los códigos, sino del campo de referencia al que cada código apunta, al “contenido” real o potencial del código. 

**Tabla 3:** Correspondencia entre subtítulos, líneas temáticas y códigos.

Subtítulo	Líneas temáticas	Códigos
Jóvenes indígenas: tensiones en la construcción de la identidad	Criterios para identificar(se) como joven indígena	Criterios de juventud
		Criterios indígenas
El entorno comunitario, las violencias vividas y la producción del malestar emocional	Condiciones de vida y su impacto en la salud-enfermedad/trastorno mental	Condicionantes de bienestar
La vida urbana y la salud mental: entre la libertad, el anonimato y la discriminación	Condiciones de vida y su impacto en la salud-enfermedad/trastorno mental	Condicionantes de sufrimiento psíquico
Caracterización de los malestares y diagnósticos asumidos	Concepciones sobre la salud y la enfermedad/trastorno mental	Concepciones salud-enfermedad mental
	Representaciones sobre el trastorno asumido	Diagnóstico asumido y manifestaciones
		Etiología asumida
		Pronóstico asumido
Formas de atención utilizadas y su pertinencia cultural	Trayectorias de atención	Identificación de anormalidad
		Inicio de búsqueda de ayuda
		Medicina tradicional*: Motivos acudir, disponibilidad y acceso, diagnóstico, tratamiento, apego y abandono, resultados, experiencia, especificidad étnica y adecuación

Fuente: Elaboración propia. * Se construyeron códigos similares a los últimos ocho para aplicarlos a cada una de las formas de atención a las que recurrieron las y los sujetos de estudio: medicina tradicional, atención biomédica, atención psicológica, atención psiquiátrica y grupos de ayuda mutua.

Las entrevistas se grabaron con autorización de las y los participantes, previa firma del consentimiento informado, y se transcribieron para su posterior codificación y sistematización con apoyo del software para el análisis cualitativo de datos Atlas.ti versión 8. Los datos se manejaron mediante pseudónimos para proteger la confidencialidad y el anonimato de los participantes. 

La observación no participante se llevó a cabo en los espacios en los que se solicitó la colaboración para establecer contacto con los actores a entrevistar. La observación se orientó al registro de elementos relacionados con la forma de representarse en los espacios institucionales a las y los jóvenes indígenas, a su salud mental, y a las maneras adecuadas de atender sus necesidades en el campo de la salud mental. A los datos derivados tanto de las entrevistas como de la observación se aplicó un análisis de contenido temático. 

El proyecto fue avalado por el Subcomité de Ética en Investigación en Salud del Centro de Investigaciones y Estudios Superiores en Antropología Social (CIESAS), Folio interno 005.

## RESULTADOS

### Jóvenes indígenas: tensiones en la construcción de la identidad

La mayoría de las y los jóvenes entrevistados cursaron estudios superiores y manifestaron tener una continua movilidad entre la Zona Metropolitana de Oaxaca y su lugar de origen (generalmente en un contexto rural o semiurbano). Esta movilidad ha implicado también una movilidad sociocultural que ha producido tensiones en la forma de concebirse a sí mismos como “jóvenes”, pues en tanto en la Zona Metropolitana de Oaxaca esta categoría está plenamente legitimada como parte del ciclo de vida, en sus lugares de origen es una categoría de emergencia reciente, a partir de la disponibilidad de niveles de educación media superior y superior, del acceso a Internet y de los procesos migratorios. 

Las y los jóvenes entrevistados han resuelto estas tensiones privilegiando e introyectando una concepción occidental de juventud y han asumido la mayoría de los rasgos que la caracterizan, tales como un estado civil de soltería, desempeñar un rol de estudiante, asumir cierta libertad y autonomía respecto a los padres o asumir el derecho a la diversión en compañía de pares. Sin embargo, esta identidad se ve cuestionada por sus familiares que permanecen en sus comunidades de origen, quienes suelen comunicarles la exigencia social de transitar a la adultez en edades en las que, en el contexto urbano, se hacen corresponder a la categoría de adolescencia (13 o 14 años). Si bien ninguna de las personas entrevistadas refirió que estas situaciones le hubieran generado un sufrimiento emocional importante, sí mencionaron cierta confusión y frustración al no lograr una completa adaptación de sus modos de vida, prácticas y experiencias subjetivas a la consecución de etapas que se asumen universales desde el planteamiento del ciclo de vida occidental.

En cuanto a su autoadscripción étnica, las personas jóvenes entrevistadas identificaron una serie de rasgos que las distinguía de las personas “mestizas”, principalmente la lengua y las costumbres y tradiciones, aunque algunas incluyeron elementos fenotípicos como el color de la piel. Sin embargo, señalaron que preferían ser identificadas con el gentilicio de su región o pueblo o con el nombre de su etnia/lengua especifica (zapoteca, mixteca, chatina, etc.), que con el concepto genérico de “indígena”. Uno de los jóvenes reflexionó sobre esta preferencia y la asumió relacionada con experiencias vividas en el contexto urbano, en las que se utilizaba el término “indígena” para manifestar burla y desprecio.

*“…tal vez en mi pasado esté esa palabra como malinterpretada, porque en realidad no es mala, yo no la considero mala ahora, pero sí cuando me decían indígena, chatino o indio en la secundaria. No sé cómo expresarlo, pienso en mi cabeza de que alguien con mayor autoridad quiera hacerme de menos cuando dice eso* [pero] *yo sé que no es una palabra mala, es un orgullo ser una persona de raíces indígenas*”. (Fernando)

Refirieron que este tipo de experiencias les produjo sufrimiento emocional; sin embargo, es preciso destacar que también en sus comunidades de origen enfrentaron situaciones que les generaron diversos grados de malestar, tal como se describe en el siguiente apartado, destacando que algunas de ellas fueron consideradas como causa de los trastornos mentales que desarrollaron. 

### El entorno comunitario, las violencias vividas y la producción del malestar emocional

Las manifestaciones de malestar emocional descrito por las y los jóvenes fueron variadas, pero tuvieron un denominador común: estar relacionadas con diferentes tipos de violencia que vivieron en sus comunidades de origen. Ismael, uno de los jóvenes entrevistados refirió que la violencia que presenció de su padre hacia su madre, cuando su padre se alcoholizaba, contribuyó al desarrollo de su “neurosis”. También Nicolás refirió que el alcoholismo de su padre y la violencia de éste hacia su madre lo había afectado emocionalmente. Si bien la violencia intrafamiliar no es exclusiva de contextos indígenas, en los relatos analizados se identificó cómo la permisividad del contexto respeto del control masculino sobre la vida de las mujeres o la existencia de matrimonios arreglados y en muchos casos forzados -rasgos característicos de algunas comunidades rurales e indígenas de México- favorecieron la producción de este tipo de violencia. 

Por su parte, Alma señaló que la *envidia* que despertaba en sus compañeros de escuela por destacar en el ámbito académico contribuyó al acoso escolar que sufrió, y que identificó como uno de los detonantes de su depresión. Señaló que este tipo de dinámicas, basadas en la envidia, constituyen uno de los rasgos culturales de su comunidad de origen. También en su caso pudo apreciarse cómo los valores comunitarios respecto a la importancia de la unión y las lealtades familiares favorecieron la impunidad ante situaciones de violencia sexual por parte de familiares.

“*Creo que la idea de familia que hay en mi pueblo hace que este tipo de cosas se encubran más. Porque incluso si tu familia sabe lo que te pasó, pero fue… no sé, tu abuelo, o tu tío, es más importante para la familia mantener el vínculo, y entonces es ‘no digas nada porque es tu familiar’*”. (Alma)

Algunas de las personas profesionales entrevistadas señalaron la importancia de los medios de comunicación en la construcción de las expectativas sobre las relaciones familiares o los modos de ser joven, lo que puede producir un malestar emocional entre quienes no pueden desarrollar los estilos de vida y formas de relacionarse que se legitiman en estos medios. Uno de los jóvenes entrevistados señaló que al comparar la imagen de familia que veía en los medios con su propia realidad familiar experimentaba frustración y que fue el inicio de una serie de insatisfacciones que lo llevaron más adelante a un consumo problemático de sustancias.

“…*aunque yo vivía en el pueblo, muchas veces no lo aceptaba, por la idea que te venden a través de la televisión de cómo es una familia perfecta* […] *tengo una imagen muy clara en mi mente de cuando yo veía la tele, y veía cómo una familia se ponía a jugar en el pasto con sus hijos, con un juego nuevo, o no sé, y pues eso me imaginaba que era una familia feliz, una familia tranquila, y en mi casa no había sino la necesidad de trabajar muy duro* [entonces] *yo empecé a rechazar, yo empecé a querer más cosas. Entonces yo por insatisfacciones yo le atribuyo que pasó esas cosas* [consumo de sustancias]”. (Fernando)

Como puede apreciarse, algunos de los rasgos de la cultura de las comunidades de origen de las y los jóvenes favorecieron la producción de violencias de diverso tipo: intrafamiliar, sexual, escolar o simbólica, con un evidente componente de género, de las cuales fueron testigos o víctimas, y que les produjeron un sufrimiento emocional significativo. 

### La vida urbana y la salud mental: entre la libertad, el anonimato y la discriminación

Solo una de las jóvenes entrevistadas (Magali) nació en la Zona Metropolitana de Oaxaca, el resto era originario de localidades rurales y tuvo diferentes experiencias migratorias: uno de los jóvenes (Nicolás) llegó siendo niño, como parte de una familia que buscaba mejores oportunidades laborales. Tres de las personas entrevistadas (Julia, Paula y Fernando) migraron durante su etapa escolar (secundaria o bachillerato) motivados por el deseo de continuar sus estudios en la ciudad, aunque en el caso de Fernando la motivación incluía aprender español y lograr cambiar sus condiciones de vida.

“*Yo decidí venir, yo insistí a mis papás, les decía que yo quería venir a estudiar acá, más que nada para cambiar, no me gustaba mucho estar en el pueblo, mi trabajo era trabajar en el campo, ir por leña, ayudar a mis papás, a meterle en la albañilería, entonces no me gustó mucho y decidí que quería aprender español, el objetivo era aprender español y también terminar una carrera* […] *para aprender más*”. (Fernando)

Otra de las participantes migró durante su etapa escolar para dejar atrás una situación de acoso escolar (Alma), y uno de los jóvenes (Ismael) llegó en busca de ayuda para su malestar. 

Como puede apreciarse, en algunos casos, el proceso migratorio incluía expectativas personales o familiares que, en términos generales, fueron alcanzadas. A excepción del joven que migró en busca de atención en un centro de internamiento voluntario gestionado por el movimiento de Neuróticos Anónimos, el resto tuvo acceso a oportunidades educativas e ingresaron o terminaron sus estudios superiores. Además, las personas jóvenes entrevistadas relacionaron la vida urbana con elementos positivos, como la oportunidad de conocer nuevos lugares y personas, salir a caminar, salir a comer, tener acceso a espacios recreativos y tener más libertad que en sus comunidades. Uno de los jóvenes aludió también a un cambio de mentalidad, pues identificó un contraste entre la mente de quienes viven en el pueblo y quienes viven en la ciudad, mostrando una valoración positiva de la mentalidad citadina.

*“…cuando uno es de pueblo la mente cerrada, de verdad yo lo digo y yo entiendo a mis papás, a mis primos, a otras personas que no han experimentado cosas de venir acá, o estar con personas con más pensamientos libres, o más liberales*”. (Fernando)

Sin embargo, mudarse a la ciudad representó también la necesidad de adaptarse a un entorno desconocido y, en ocasiones, intimidante. En términos prácticos, como parte de los retos cotidianos, mencionaron aprender a desplazarse en la ciudad o usar el trasporte público sin perderse, o aprender los códigos de interacción locales. También señalaron dificultades para adaptarse a las formas de relacionarse, identificando una diferencia sustancial en el tipo de sociabilidad que se produce en la ciudad y en sus pueblos. Uno de los jóvenes señaló que en la ciudad

“*…como que cada quién está buscando lo suyo* […] *y allá* [en su pueblo] *es como… se hace como más la unidad, vamos a ir a traer la lluvia… para que llueva y crezcan las plantas, crezca el maíz*”. (Fernando)

También referido al ámbito de las relaciones sociales, algunas personas participantes compartieron la sensación de anonimato en la ciudad, la cual contrastaba con el reconocimiento mutuo en sus comunidades. 

La actitud de los citadinos hacia lo indígena/rural (binomio que en el imaginario de las personas entrevistadas parece estar íntimamente ligado) marcó su experiencia en la ciudad. Oaxaca de Juárez (capital del estado) se presenta como una ciudad multicultural, su máxima fiesta y sus atracciones turística exaltan y celebran la diversidad étnica. Sin embargo, se identificó una actitud ambigua hacia lo indígena caracterizada por la admiración y valoración de ciertos elementos que se asocian con las culturas indígenas a la vez que el desprecio y rechazo hacia algunos de los portadores de dichas culturas, sobre todo cuando se intersectan (realmente o en el imaginario) con condiciones de pobreza y marginalidad. Estas contradicciones fueron explicadas en los siguientes términos por uno de los profesionales entrevistados:

“*Depende de la temporada, porque en el mes de la Guelaguetza los aceptan mucho* [a los indígenas], *pero en otras temporadas la cuestión indígena se ha visto como un medio de discriminación. Te discrimino por venir de una comunidad, porque no llevas un calzado, porque a lo mejor no estás bien vestido, porque no traes una ropa normal, sino traes la ropa de tu región. En julio es bien visto, entonces se toman fotos, pero es más como… yo persona privilegiada estoy teniendo la oportunidad de convivir contigo, soy una mejor persona porque estoy conviviendo contigo*”. (Psicólogo servicio público)

Esta ambigüedad se expresó también en el entorno escolar donde vivieron experiencias de reconocimiento y valoración de sus rasgos culturales (principalmente su lengua), a la vez que rechazo y burlas por este y otros marcadores, como el color de piel o la condición de “pueblerino o pueblerina”, lo que les produjo malestar emocional y los llevó a tratar de ocultar o disimular estos marcadores, por ejemplo, evitando hablar su lengua en público.

“*Se fueron enterando* [los compañeros de la secundaria] *que yo hablaba una lengua indígena* […] *Todavía me acuerdo de la palabra que me dijo, me dijo ‘india’* […] *y en el bachillerato, ya que empecé a usar redes sociales, me acuerdo de que en una red social una cuenta anónima me puso algo así de que ‘regrésate a tu cerro con tus nopales y tus burros’. Eran muy despectivos los comentarios*”. (Paula)

Cabe destacar que en el contexto urbano las personas jóvenes entrevistadas también encontraron relaciones amables y significativas que las ayudaron a integrarse a la vida en la ciudad y que representaron un importante apoyo emocional. No obstante, las experiencias de acoso, entendido como comportamientos hirientes, repetidos y en el contexto de diferencias de poder entre acosadores y víctimas^60^, produjeron un sufrimiento psíquico que generalmente se vivió en silencio, sin comunicarlo a profesores o familiares. Uno de los jóvenes compartió que nunca habló con nadie sobre el acoso que sufría porque pensaba que sus profesores lo verían con lástima y que su familia, al compartir la condición objeto de desprecio, iba a sentirse lastimada:

“*…dolía cuando te decían eso, es como una herida fuerte, y entonces si yo le preguntaba a mi hermano: ‘¿cómo ves esto?’, yo pienso que* [para él] *era una herida fuerte*”. (Fernando)

Las y los jóvenes aludieron a situaciones que, sin estar directamente relacionadas con su condición étnica, fue posible identificar cómo esta condición le imprime a las situaciones características específicas. Por ejemplo, si bien el estrés escolar es una condición que puede presentarse sin distinción de etnia o contexto de residencia, algunas de las personas entrevistadas señalaron percibir una desventaja en su formación académica respecto a sus compañeros que habían cursado los niveles previos en contextos urbanos. De manera similar, la búsqueda de un sentido de pertenencia puede considerarse común en los jóvenes, lo que puede favorecer el inicio en el consumo de sustancias, tal como expresó Fernando: “la primera vez que consumí marihuana fue como para estar igual con la otra persona que convives, para sentirme bien, para sentirme parte”.

Algunos de los jóvenes entrevistados refirieron que el rechazo experimentado por su condición étnica hizo, en algunos casos, más urgente esta necesidad de reconocimiento y aceptación, además de que la legitimación de un consumo “fuerte” de alcohol en sus pueblos contribuyó a que se iniciaran en esta práctica. Sin embargo, la relación entre las experiencias de rechazo, lo que ellos denominaron depresión y el consumo de sustancias no la asumieron necesariamente como unidireccional ni configurando una cadena causal, pues, en algunos casos, lo que identificaban como depresión se presentaba previo o a la par de su consumo, consumían sin que mediara depresión, e incluso concebían el consumo de sustancias como una forma de automedicación.

“*Yo con la primera vez que consumí fue una sensación muy agradable, una sensación de risa, de felicidad muy al extremo, una felicidad inmensa, y cada vez que consumía buscaba regresar a ese grado de felicidad* [pero] *también lo que puedo detectar es de que consumo por todo y por nada, porque estaba yo triste, porque estaba yo enojado, porque estaba yo ansioso, porque tenía hambre, porque no tenía hambre, por todo, porque ganó México, porque perdió México*”. (Fernando)

### Caracterización de los malestares y diagnósticos asumidos

En el momento de las entrevistas solo una de las participantes utilizó una categoría nosológica propia de la medicina tradicional como su diagnóstico asumido (aunque al mismo tiempo utilizaba la categoría de “trauma”). El resto de las y los jóvenes utilizaron categorías propias de la psicología y psiquiatría para nombrar e interpretar su condición (Tabla 4). 

**Tabla 4:** Diagnóstico asumido por las y los participantes del estudio. Zona Metropolitana de Oaxaca, México, 2023.

Pseudónimo	Diagnóstico asumido	Tipo de diagnóstico
Julia	Trauma/espanto	Autodiagnóstico
Paula	Síndrome conversivo/ansiedad	Psicológico
Magali	Ansiedad	Autodiagnóstico
Alma	Trastorno de ansiedad generalizada con ataques de pánico	Psiquiátrico
Nicolás	Alcoholismo	Autodiagnóstico
Fernando	Depresión y alcoholismo	Autodiagnóstico
Ismael	Neurosis	Autodiagnóstico

Fuente: Elaboración propia con base en los datos recabados.

De acuerdo con las personas jóvenes entrevistadas, las diversas experiencias desagradables que vivieron fueron produciendo condiciones emocionales y comportamentales que en algún momento identificaron como patológicas. En los cuadros incluidos en el Material Suplementario se describe el malestar o sufrimiento reportado por cada una de las personas participantes. Tristeza, depresión, llanto, sensación de soledad, miedos irracionales, agresividad, ansiedad y su somatización, ataques de pánico, consumo problemático de sustancias e intentos de suicidio formaron parte de los síntomas y problemas señalados. Durante las trayectorias analizadas, las y los jóvenes fueron nombrando y representando a su condición de diferentes maneras. 

El hecho de que solo dos de las personas entrevistadas asumieron un diagnóstico propio de la psicología o la psiquiatría al momento de la entrevista, no implicaba que el resto de las y los jóvenes no accedieran en algún momento de su trayectoria a este tipo de servicios. De hecho, solo uno de los jóvenes no procuró atención psicológica (de un profesional titulado) y solo habló con una estudiante de psicología que estaba haciendo sus prácticas. Esta situación se explica por otras vías. Por una parte, de acuerdo con los relatos de las personas entrevistadas, desde algunas corrientes psicológicas cuestionan la importancia de establecer un diagnóstico y prefieren trabajar sobre los problemas y no etiquetar a sus consultantes. Por otro lado, dos de los jóvenes que sí accedieron a atención psicológica mencionaron que esta no les resultó significativa ni útil porque la psicóloga no comprendía su problema, o porque tuvieron reticencia a contarle su vida.

“*Yo creo que ya no fui* [a una segunda sesión con la psicóloga] *porque me vi amenazado de que alguien supiera como había sido mi vida y los problemas que en ese momento tenía, no quería que alguien viera realmente lo que era yo, lo que estaba sucediendo en mi vida*”. (Nicolás)

Entre las personas entrevistadas cabe destacar una preferencia de las mujeres por la atención psicológica, lo cual coincide con el señalamiento de una de las psicólogas entrevistadas de que la mayoría de sus consultantes son mujeres; y una preferencia de los tres varones por los grupos de ayuda mutua como forma de atención significativa. Dado que en estos espacios no participan terapeutas profesionales, los varones asumieron el alcoholismo, la depresión y la neurosis como autodiagnósticos. 

### Formas de atención utilizadas y su “pertinencia cultural”

Las trayectorias de atención seguidas por las y los jóvenes mostraron gran diversidad en términos de duración, formas de atención utilizadas, orden de utilización de estas, experiencias durante la atención y eficacia percibida de dichas instancias. 

Como se mencionó previamente, se considera que una trayectoria de atención se inicia al momento en que los sujetos identifican una condición de anormalidad. En la mayoría de los casos estudiados, esta identificación de la “sintomatología” se produjo de manera retrospectiva, una vez que se habían apropiado de los modelos etiológicos propuestos por las opciones terapéuticas a las que habían recurrido durante su trayectoria y que les habían resultado significativas. Ante la identificación de la anormalidad, las primeras prácticas que realizaron las y los jóvenes correspondieron a lo que Menéndez denomina el modelo basado en la autoatención^61^. 

#### Prácticas de autoatención

En la mayoría de los casos las y los jóvenes refirieron haber desarrollado diversos procesos de autoatención, entendida esta como los saberes que los sujetos y microgrupos retoman de otras formas de atención y los utilizan, en términos generales, para asegurar la reproducción biosocial de los sujetos y microgrupos y, en un sentido acotado, para

“…diagnosticar, explicar, atender, controlar, aliviar, soportar, curar, solucionar o prevenir los procesos que afectan su salud en términos reales o imaginarios, sin la intervención central, directa e intencional de curadores profesionales, aun cuando éstos pueden ser la referencia de la actividad de autoatención”.^61^

Cabe destacar que este tipo de prácticas no se limitó a los momentos previos a la primera búsqueda de ayuda profesional, sino que se continuaron realizando durante toda la trayectoria, como la única forma de atención en un momento determinado, o a la par de otras formas de atención. Algunas de estas prácticas las desarrollaron de manera intuitiva (como tomar un baño para calmar la ansiedad), algunas se basaron en información encontrada en Internet, por ejemplo, sobre ejercicios de meditación y *mindfullness*, y algunas se derivaron de recomendaciones de profesionales consultados previamente. Algunas de estas prácticas fueron de tipo colectivo, por ejemplo, la participación en grupos de apoyo en Facebook, o acciones de la familia, como la ayuda práctica en las labores cotidianas, consejos o apoyo emocional. Cabe destacar que este tipo de prácticas se presentaron en mayor medida en las jóvenes, en tanto que entre los varones la forma de autoatención más destacada fueron los grupos de ayuda mutua sobre los que se profundizará más adelante. Se destacan también diversas prácticas de automedicación relativas al consumo -no necesariamente considerado problemático- de tabaco, alcohol y otras drogas, como una forma de aliviar los malestares. 

Dos personas reconocieron y valoraron las prácticas rituales realizadas en familia como un recurso para la autoatención que, si bien no se realizaban para curar o prevenir alguna dolencia en particular, sí se orientaban a favorecer la salud (entre otros bienes valorados) o como una experiencia espiritual. Fernando ha participado, junto con su familia, en cultos a los Dioses y a la Madre Tierra, encontrando similitudes de estas prácticas con las enseñanzas de la Biblia y del programa de Alcohólicos Anónimos. Paula, por su parte, señaló que en los rituales que lleva a cabo su familia se articulan elementos “de los ancestros” y elementos del catolicismo y reconoció una eficacia simbólica derivada de una decisión personal sobre lo que creer, como un acto de fe.

“*Cuándo pasé lo de la amenaza de aborto, hubo tiempo en el que como que me quería agarrar otra vez la ansiedad, de sobre pensar demasiado las cosas, imaginarme los peores posibles escenarios, pasé por eso, pero en ese entonces mi mamá empezó a hacer estos rituales de ir a las casas de los rayos, entonces aunque yo no estuviera presente, de cierta forma como que me confortaba el saber que ella lo estaba haciendo* […] *no tanto como eficacia médica, sino que uno como persona se dé cuenta de que creer en algo, tener fe en algo puede ayudarte* […] *creo que es no tanto como eficacia médica, sino como cuestión personal* […] *en este caso como yo metí ahorita más mi fe a la parte de los rituales indígenas que tenemos allá en la comunidad, como que me ha ayudado mucho, como que personalmente me da mucha fortaleza, pero es una decisión que decidí tomar yo*”. (Paula)

La búsqueda de ayuda profesional se inició ante una diversidad de circunstancias, entre ellas, un malestar físico evidente, la incapacidad para llevar a cabo sus actividades cotidianas, o un sufrimiento emocional que se tornó insoportable. Esta búsqueda de ayuda estuvo condicionada, de acuerdo con los relatos de las personas participantes, por los recursos terapéuticos disponibles en el entorno, los recursos económicos personales y familiares, las experiencias previas (positivas o negativas) con opciones terapéuticas específicas, el impacto del trastorno en su vida y los modelos explicativos de la enfermedad que los sujetos y/o sus padres suscribían en cada momento de la trayectoria. En los siguientes apartados se describen algunos aspectos relacionados con las diferentes formas de atención utilizadas por las y los jóvenes. El orden de presentación no corresponde a un patrón de orden cronológico de utilización en las trayectorias analizadas, patrón que no es posible identificar pues los caminos recorridos de las personas participantes fueron heterogéneos. 

#### La utilización de la medicina tradicional

La medicina tradicional fue una de las formas de atención utilizadas principalmente cuando las y los jóvenes se encontraban residiendo en sus pueblos. Su utilización estuvo relacionada con su disponibilidad y facilidad de acceso en términos de cercanía, costos y horarios, así como con la confianza propia y/o de sus familiares en su eficacia. Cuando residían en el contexto urbano su utilización fue limitada, a pesar de estar disponible. 

El susto fue la categoría nosológica tradicional más mencionada por las y los participantes y varios de ellos asumieron al menos la posibilidad de haber presentado los signos. Dos jóvenes refirieron haber acudido a un curandero para tratar el susto, derivado principalmente de accidentes, con buenos resultados terapéuticos. 

Las curanderas entrevistadas explicaron la condición de espanto o susto como resultado de la separación del alma de su cuerpo físico y explicaron que la terapéutica se orientaba a lograr el regreso del alma a su cuerpo. Por su parte, las y los jóvenes que buscaron atención tradicional para tratar el susto hicieron énfasis en los procedimientos de cura y solo ante la solicitud explícita se refirieron a las creencias que orientaban esas prácticas. Respecto a la eficacia terapéutica de las “limpias” para tratar su condición de susto o espanto, Fernando afirmó que fue eficaz, en tanto que Julia reconoció la posibilidad de una eficacia real fundamentada en su compatibilidad con el conocimiento científico.

“*Hay ciertas cosas que creo que sí ayudan, por ejemplo, algunas plantas medicinales son muy benéficas porque en realidad los fármacos y demás vienen de las plantas, de la naturaleza… siento que sí creo en esas cosas… son compatibles*”. (Julia)

Otra de los trastornos abordados por las y los jóvenes fue el mal producido por envidia, que algunos refirieron como brujería. Respecto a este constructo, Julia reconoció la posibilidad de que las personas puedan provocar un mal a otras por envidia pues “*como existen pensamientos buenos también existen pensamientos malos*”; sin embargo, limitó esta posibilidad al hecho de que la persona hacia la que va dirigido el mal efectivamente crea que puede ser afectada, y que la persona que desea ese mal sea alguien cercano. Al respecto señaló:

“…*yo digo, por más que me haga* [brujería] *no va a pasar nada porque no me está dando de comer, no se preocupa por mí, no está viendo por mí, yo soy la que anda apurada o agitada en mis cosas*”. (Julia)

También señaló que esto difiere de las creencias de otras personas de su comunidad, que consideran que la envidia las puede afectar, sin mediar las consideraciones mencionadas. 

Si bien en la mayoría de las y los jóvenes no se produce una desacreditación explícita de la medicina tradicional, ni se niega de manera determinante su potencial eficacia, en las jóvenes con formación universitaria en el área de la salud se identificó cierta toma de distancia respecto a la utilización de la medicina tradicional, poniendo en duda su eficacia respecto de ciertas condiciones y prácticas (psicosis, suicidio) y privilegiando concepciones y abordajes de la medicina científica, evidenciando la introyección de una lógica cientificista.

“*El hermano de mi pareja tiene psicosis, algo así, pero es por estrés* […] *sus papás me dijeron ‘es que se volvió loco porque lo embrujaron, porque nos odian mucho’* […] *y lo llevaban* [con un brujo] *y él cada vez estaba más mal* […] *yo siempre les decía ‘es que sí es bueno que le estén haciendo curas, que pongan esas velas o entierren un candado, pero también hay que apoyarnos en la ciencia’. Ya cuando vieron al límite la situación de que se estaba poniendo agresivo fue cuando me dijeron ‘sí, vamos a llevarlo’. Tienen que llegar a un cierto punto para que acepten la ciencia o acepten otra forma que no sea de su mundo*”. (Julia)

Pareciera también asumirse que en estos casos se asume un pensamiento de corte evolucionista, en donde se considera que el momento de aceptar la noción biomédica de la enfermedad mental está por llegar a las comunidades, lo cual es deseable, pues correspondería a una aproximación correcta a la realidad. Una de las jóvenes relacionó explícitamente esta forma de pensar con su formación profesional.

“*Como que* [en las comunidades] *aún no está esta idea de que hay patologías que afecten a la mente y que la persona pudiera estar sana físicamente, pero mentalmente no* […] *en caso de que alguien se haya suicidado, como que no se tenía esa noción de un padecimiento mental, que en este caso podría ser la depresión. Entonces lo primero que decían era ‘es que no quiere trabajar’ o ‘se quitó la vida porque no quiere mantener a sus hijos’* […] *pero, en realidad era eso,* [un trastorno mental] *pero yo creo que mi mentalidad cambió desde que empecé a estudiar enfermería*”. (Paula)

En los casos de trastornos depresivos, ansiosos y consumo de sustancias que configuran una trayectoria larga (trayectorias mayores o iguales a cuatro años), algunas de las y los jóvenes señalaron haber acudido en su etapa infantil o adolescente a un curador tradicional por decisión de sus padres, quienes asumieron que su mal podría tratarse de un espanto o brujería, pero señalaron que no les resultó eficaz para tratar su padecer.

Como puede apreciarse, es posible señalar que las y los jóvenes realizaron prácticas de atención basadas en la medicina tradicional, aceptaron la existencia de algunas de sus categorías nosológicas, pero establecieron distancia respecto a la forma en que se concibe su etiología en su comunidad. Respecto a la eficacia de estos recursos terapéuticos se identificó que en algunos casos explicaron la posibilidad de una eficacia real, recurriendo a los fundamentos del conocimiento científico. 

### Formas de atención occidentales

La residencia en un contexto urbano favoreció la disponibilidad de diferentes formas de atención. El dominio del español, la utilización de las tecnologías de la información y comunicación (centralmente Internet), un nivel educativo mayor al básico (en la mayoría, incluso, nivel superior), contar con una forma de aseguramiento o con recursos personales o familiares favoreció el acceso a las consultas privadas pagas. En el entorno urbano las y los jóvenes entrevistados refirieron haber utilizado servicios de medicina general, psicología, psiquiatría y de grupos de ayuda mutua. En este apartado se presentan algunos de los hallazgos, centrándonos en la adecuación cultural de las opciones terapéuticas utilizadas. Cabe señalar que, en la mayoría de los relatos, este tema no emergió de manera espontánea como una barrera de acceso o un motivo de insatisfacción; sin embargo, cuando se planteó el tema, las personas entrevistadas relataron diversas situaciones relacionadas.

#### La atención biomédica

Las y los jóvenes refirieron haber recurrido al médico general o a los servicios de urgencias cuando su enfermedad se atribuyó a un trastorno orgánico, o ante un evidente deterioro físico derivado del malestar emocional. Esta forma de atención no resultó significativa para la atención del malestar de las y los jóvenes; sin embargo, contribuyó a descartar un problema orgánico y a orientar la trayectoria hacia los servicios de psicología mediante la recomendación o la derivación formal. 

#### La atención psicológica

En el entorno urbano, la atención psicológica estuvo disponible y, desde el punto de vista de las y los entrevistados, resultaba accesible tanto en los servicios públicos como en los privados, pues en estos últimos el costo por consulta oscilaba entre $100 y $500 pesos mexicanos (entre $7 y $30 dólares), lo cual no representaba una barrera económica, aunque en algunos casos sí representó un gasto que se salía del presupuesto mensual. Seis de los jóvenes acudieron al menos una vez a atención psicológica durante su trayectoria. 

Los servicios psicológicos a los que accedieron las y los jóvenes tuvieron enfoques diversos. A partir de sus relatos, se pudieron identificar intervenciones con tintes religiosos, intervenciones centradas en ofrecer estrategias para el control del estrés, otras centradas en motivar a las personas consultantes, o psicoterapia con enfoque de género e interseccional (siendo éste el más apreciado por las jóvenes entrevistadas). Las y los psicólogos entrevistados corroboraron que existe una diversidad de enfoques dentro de su disciplina. 

En cuanto a la pertinencia cultural de los servicios, entre algunos de los profesionales de esta disciplina se identificó una representación de los indígenas basada solo en el criterio lingüístico o el lugar de origen, pero desconociendo la variedad de criterios utilizados por las y los jóvenes para configurar su identidad étnica. Una de las psicólogas ubicó este referente identitario en el pasado “*porque indígena, la raza, raza en sí de indígena, ya ni existen, todos somos mezcladitos*” (Conversación con psicóloga durante el desarrollo de la observación) 

Se identificó también entre algunos profesionales una imagen estereotipada del indígena como aquel que habita en contextos rurales y marginados, es pobre y tiene bajos niveles educativos. Sin negar que esta es una realidad para la mayoría de la población indígena en México, estas nociones evidencian el desconocimiento de la diversidad -y desigualdad- al interior de este colectivo. 

La percepción de aceptabilidad cultural de esta forma de atención fue variable. En algunos casos, las jóvenes identificaron cierta distancia entre las recomendaciones de las y los profesionales y la posibilidad de llevarlas a cabo dada la cultura propia.

“*Me atendieron como una persona más que vivía en una población urbana* […] *no tomaron en cuenta que yo venía de un contexto indígena y que las situaciones en cuestión de patologías mentales son muy invisibilizadas, y que el hecho de que estallara mi ansiedad por no expresar mis sentimientos y emociones, pudieran estar relacionadas con el contexto en que crecí* […] *en ese tipo de culturas es muy difícil que la gente exprese lo que siente, sus sentimientos y sus emociones libremente*. [Es difícil] *que los papás te hablen sobre aspectos de salud mental, sobre la confianza, sobre platicar problemas* […] *el hecho de que yo no pueda expresar mis sentimientos es algo con lo que vengo creciendo, entonces creo que cuándo me dan estas estrategias de ‘háblalo, coméntalo’, como que no tenían en mente que se me iba a hacer complicado*”. (Paula)

No obstante, tanto jóvenes como profesionales señalaron que la psicología dispone de una variedad de encuadres teóricos, y que los abordajes contextuales, interseccionales o narrativos cuentan con herramientas para abordar la diversidad cultural y la desigualdad social, partiendo de la aceptación de que las características sociales y fenotípicas dan lugar a formas diferentes de opresión y, por ello, a necesidades específicas de atención.

“*Ella* [la psicóloga que la atiende] *está consciente de que las mujeres somos diferentes y que dependiendo de nuestra situación tenemos necesidades diferentes, entonces nunca he sentido que la teoría de la que ella me habla sea solo para mujeres blancas, o que yo no lo pueda aplicar por la… el contexto en el que me desarrollo, o que yo tenga un requerimiento como de ‘tienes que hacer esto y esto’ que parezca… o sea que sea imposible de hacer* […] *es como ubicar los tipos de opresión o discriminación que cada persona vive tomando en cuenta sus propias características, o sea una persona indígena no vive el mismo tipo de opresión que una persona blanca, una mujer negra no vive el mismo tipo de opresión que una mujer… no sé, europea que tiene dinero, es como ubicar nuestras características sociales, físicas, o sea todo lo que te construye como una persona y entender que a partir de eso tú sufres de cosas diferentes*”. (Alma)

Se destaca que, en algunas trayectorias, las personas jóvenes recurrieron más de una vez a la atención psicológica y las experiencias fueron diferentes en función del enfoque y la personalidad de quien los atendía. Tanto mujeres como varones señalaron que algunas de las personas con las que se atendieron no los comprendían, en tanto que las jóvenes mencionaron que con algunas o algunos terapeutas sí lograban establecer una relación basada en la escucha y la confianza. En algunos casos, sobre todo entre las mujeres, esta forma de atención se reconoció como eficaz, asumiendo su diagnóstico, aunque generalmente reinterpretado. En otros casos, principalmente entre los varones, no lograron confiar en sus terapeutas y externarles su vida y sus problemas, no les resultó eficaz esta forma de atención, y los sujetos continuaron su búsqueda de alivio en otras instancias.

#### La atención psiquiátrica

En la Zona Metropolitana de Oaxaca la atención psiquiátrica estaba disponible, aunque podría decirse que su costo de entre $700 y $1.000 pesos mexicanos por consulta (entre $40 y $60 dólares), podría significar una limitante para el acceso efectivo. Solo una de las jóvenes entrevistadas utilizó este tipo de atención (y una de las responsables de grupos de ayuda mutua entrevistadas, durante su juventud). Ambas utilizaron servicios privados, a pesar del gasto que esto representó. En ambos casos se recurrió a esta forma de atención por recomendación de otro tipo de terapeuta (médico o psicólogo). Quienes recurrieron a esta forma de atención coincidieron en identificar, como uno de sus rasgos, el abordaje farmacológico basado en un diagnóstico centrado en la dimensión orgánica. 

La experiencia relatada por las entrevistadas respecto a esta forma de atención fue variada y se incluyeron algunos ejemplos de inadecuación del abordaje terapéutico respecto a sus condiciones de vida; sin embargo, esto no estuvo relacionado con su nivel de satisfacción ni con su decisión de continuar o abandonar el tratamiento. 

La psiquiatra entrevistada compartió situaciones en las que identificó la importancia de la cultura para la construcción de la enfermedad mental y su adecuado diagnóstico; sin embargo, reconoció que en su formación de especialidad no le brindaron herramientas para abordar este tema.

“*Una señora aseguraba que en su casa había espíritus, bueno, en mi evaluación psiquiátrica debo descartar que esté psicótica, pero resulta que toda la familia y en la comunidad es parte de la creencia que hay espíritus, incluso se les venera, se les tiene ciertas consideraciones, y eso es normal. Para otra persona podría pasar como que sí tiene un trastorno psicótico… creo que se conoce muy poco sobre qué adaptaciones se deberían hacer*”. (Psiquiatra práctica privada)

Dicha profesional, aunque reconoció el rol de lo social en la construcción del padecimiento psiquiátrico, afirmó que solo una práctica clínica multidisciplinaria, en donde la psicología se encargara del abordaje de lo social, posibilitaría una atención que integrara los aspectos culturales del paciente. 

La joven que recurrió a esta forma de atención tuvo experiencias diferentes con dos psiquiatras. El primero al que consultó (un varón) por iniciativa de su madre le comunicó desde la primera cita su diagnóstico y le indicó medicación. Su experiencia con este psiquiatra fue negativa, pues el diagnóstico la asustó y los medicamentos le provocaron fuertes efectos secundarios.

“*Él me dijo que yo tenía un trastorno de ansiedad generalizada con ataques de pánico, y pues al principio el diagnóstico asusta mucho porque yo no tenía la idea de nada, entonces eso me hizo sentir mal también, y luego vino el medicamento que creo que nunca se reguló de manera correcta para mí porque pues cuando empecé a tomarlo pues era muy joven, tenía diecinueve, y entonces me sentía como muy fuera del mundo, no entendía cuando me hablaban, y después de un tiempo cuando yo hablaba decía las cosas en desorden, o decía cosas que no tenían nada que ver con la conversación* […] *no tenía emociones, ya no lloraba ni me quería matar, pero no hacía otra cosa*”. (Alma)

Ante esta reacción a los medicamentos y el hecho de que el psiquiatra no respondiera sus llamadas, sus padres decidieron que abandonara el tratamiento. Años después acudió con otra psiquiatra, por recomendación de su psicóloga tratante, quien confirmó el diagnóstico del psiquiatra previo, agregó “*episodios de trastorno obsesivo compulsivo*” y también le prescribió medicamentos. Sin embargo, la experiencia en esta ocasión fue positiva, pues la médica le explicó las razones para medicarla, los posibles efectos de los medicamentos, y estuvo monitoreando su reacción a la medicación para ajustarla, además, refirió que la atención articulada de la psicóloga y la psiquiatra le estaba dando buenos resultados. 

#### Grupos de ayuda mutua

Los grupos de ayuda mutua son agrupaciones de personas que asumen compartir una problemática similar y que se reúnen para compartir sus experiencias, basados en un programa estructurado, y el que no participan de manera directa profesionales. En la Zona Metropolitana de Oaxaca los grupos de Alcohólicos Anónimos y de Neuróticos Anónimos son una opción disponible, aunque también pueden encontrarse en el medio rural. Para integrarse a un grupo de este tipo no se requiere hacer un pago o realizar trámites, lo que favorece su accesibilidad.

Los tres jóvenes varones entrevistados refirieron encontrar en estos espacios conocimientos y dinámicas que les fueron útiles para lograr sus objetivos en cuanto al control de su enfermedad, destacando la comprensión encontrada, el respeto de los compañeros, y la posibilidad de expresarse libremente en un marco de confidencialidad. Solo una de las jóvenes utilizó una modalidad de esta forma de atención que se denomina “Cuarto y quinto pasos”, y que consiste en un retiro voluntario por algunos días. Sin embargo, consideró que esta fue una mala experiencia pues refirió que los participantes compartían sus vivencias con gritos y groserías, que las actividades realizadas como parte de la terapia le produjeron miedo y que no se sintió identificada con los demás participantes pues hablaban de experiencias ajenas a ella (como haber cometido delitos o participar en el narcotráfico).

“*Es como un retiro, te llevan a un lugar en el que no hay nada, y te llevan con los ojos vendados para que tú no sepas en dónde estás, te convencen de que te van a meter a un lugar primero en donde hay perros bravos, escuchas los sonidos de los perros en la noche en medio de la nada y tienes mucho miedo, después cuando entras solo hay unas mesas pero antes de eso firmas un consentimiento de que tú quisiste hacerlo, hay unas mesas en donde los sientan a todos, te dan unas hojas y un lápiz y entonces empiezan a entrar… o sea son como temas, ahorita vamos a hablar de los celos, no sé, del abuso, entonces entra una persona que ya estuvo en el retiro y que ahora forma parte de los ponentes y te empieza a hablar de su historia pero o sea con palabras… no sé o sea todo fuera de empatía, con groserías, con gritos, como que es gente que no ha abordado los problemas y piensa que haciéndolo así va a conseguir algo, pero todavía tienen crisis hablando de eso enfrente de ti, y gritan y lloran y… pues como es gente en realidad adicta la que va ahí pues han hecho cosas muy raras*”. (Alma)

En cuanto al tema de la pertinencia cultural, en el marco de estos grupos de ayuda mutua, se atribuye a la enfermedad que tratan un carácter universal. Se asume que, aunque las manifestaciones concretas puedan variar, la enfermedad es la misma, por lo que no se requeriría adecuar la terapia para la atención de grupos sociales particulares, como pueden ser las y los jóvenes indígenas. A lo sumo se requeriría traducción cuando exista la diferencia del idioma. En sintonía con esta lógica, las y los jóvenes no señalaron aspectos que afectaran la pertinencia cultural de la terapia, solo uno de ellos comentó que algunas de las experiencias cotidianas que relataba en las juntas no encontraban eco en sus compañeros de grupo pues no tenían experiencias al respecto (por ejemplo, cuando compartía situaciones relacionadas con el cultivo de la tierra); sin embargo, no consideró que esta situación impactara en la eficacia de la terapia. Otro de los jóvenes consideró que en el grupo de su pueblo sí se hacen adecuaciones, tanto relacionadas a la lengua, como en relación con diferencias culturales o formas de pensar. Sin embargo, consideró que en el grupo al que asiste en la ciudad no es necesario realizar adecuaciones culturales.

“*…ahí en el pueblo, por el grupo en que estoy, me doy cuenta de que uno, pues, se adecúa, porque ellos hablan chatino, hay que hablar chatino, y entender que su manera de pensar es diferente, o pensamos diferente cuando estamos en el pueblo, -pues hoy me tocó ir al cerro a hablarle a Dios-, y acá no* […] *porque es diferente la cultura*”. (Fernando)

Por otra parte, algunos de los jóvenes destacaron como un aspecto positivo la similitud entre la concepción del alcoholismo que se maneja en Alcohólicos Anónimos -como una enfermedad física, mental y espiritual- y la forma de concebir a la persona en sus comunidades como un ser físico, social y espiritual. Otro elemento que parece favorecer la aceptabilidad cultural de esta terapia es el hecho de basarse en compartir experiencias de vida, que corresponderían a la realidad (cambiante y culturalmente diversa) de los diferentes colectivos en donde se materializa esta forma de atención.

## DISCUSIÓN

Los hallazgos de este trabajo coinciden con los abordajes de la *juventud* en tanto construcción histórica que alude a una clasificación social y a una identidad^62^. Dicha construcción responde, en las y los sujetos de estudio, a una forma occidentalizada de representarse y vivir la juventud y que puede entrar en conflicto con las concepciones sobre el ciclo de vida que priman en los contextos rurales y semiurbanos de donde son originarias. También la identidad étnica la viven de manera ambigua y conflictiva, en estrecha relación con las actitudes, también ambiguas, de un entorno urbano en el que puede convivir un reconocimiento-desconocimiento de las culturas indígenas, y una celebración-menosprecio de estas. 

Las y los jóvenes se asumieron como indígenas, utilizando el criterio de la autoadscripción, haciendo referencia a tradiciones y costumbres compartidas con su comunidad y destacando, como en trabajos previos^63^, la importancia del sentido de pertenencia a un grupo definido en términos étnicos, que puede ser la familia, los parientes o la comunidad. Similar a lo identificado entre estudiantes de secundaria de Oaxaca^64^, las y los jóvenes asumieron una identidad étnica basada en la diferencia cultural, pero manifestaron incomodidad respecto al término “indígena”, al que relacionaron con experiencias de rechazo y desprecio en el entorno urbano. 

Estudios previos han identificado el impacto de la experiencia migratoria en la salud mental de personas indígenas debido al contacto con una cultura ajena, la discriminación y la situación marginal en la que suelen insertarse en las ciudades^16^^,^^19^^,^^64^^,^^65^^,^^66^^,^^67^. Sin embargo, en este trabajo se pudo identificar que el trastorno mental, de acuerdo con los relatos de las y los jóvenes participantes, se construyó a lo largo de toda la trayectoria vital, incluyendo sus experiencias en sus comunidades de origen, por lo que se exploró el rol de las culturas indígenas en la producción de afectaciones a la salud mental. 

Una forma de caracterizar estas culturas es mediante la categoría de comunalidad, que hace referencia a una forma de organización social basada en las relaciones de parentesco, la reciprocidad, las formas de trabajo comunal y el control real o simbólico de la desigualdad^68^. Se reconoce que estos aspectos pueden contribuir al bienestar colectivo, pero se plantea que pueden también impactar negativamente en el bienestar individual a través de procesos como la envidia, en tanto mecanismo de control social^69^, que produce un tipo particular de acoso escolar, o la valoración de la unión familiar que puede favorecer la impunidad ante la violencia sexual al interior de la familia. Estos hallazgos coinciden con estudios previos^18^^,^^70^^,^^71^^,^^72^ que han señalado el impacto negativo que algunas prácticas tradicionales -como el matrimonio infantil forzado o la mutilación genital femenina- y otras formas de violencia (física, psicológica, económica y sexual) tienen en la vida de niñas y mujeres indígenas, y en su salud sexual, reproductiva y mental. Se plantea que no reconocer estos procesos podría configurar una forma sutil de racismo, al atribuir a los pueblos indígenas estereotipos que impiden asumir su complejidad. 

Al integrarse a la ciudad, las y los jóvenes enfrentaron un contexto sociocultural diferente y tuvieron que hacer adaptaciones de índole pragmático y simbólico. Desde autores clásicos se ha visto a la ciudad y su forma de vida como una amenaza para la salud mental de quienes la habitan. Para Kraepelin, desde un abordaje organicista, las condiciones de la vida moderna en las grandes urbes provocaban la degeneración de los individuos y de las razas, y una de las manifestaciones de esta degeneración eran precisamente las enfermedades mentales^73^. Desde un enfoque más sociológico, Simmel atribuía a la vida citadina de principios del siglo XX la capacidad de producir transformaciones importantes en el espíritu de los sujetos y analizaba los contrastes entre la vida en el campo y en las ciudades^74^. Más de un siglo después, la esencia de los contrastes señalados por Simmel parece seguir vigente y reflejarse en la experiencia de las personas entrevistadas, quienes identificaron oposiciones entre la vida en sus pueblos y en el entorno citadino de la Zona Metropolitana de Oaxaca. Para ellos y ellas, la ciudad fue sinónimo de libertad, rapidez y anonimato, mientras que en su pueblo se vivía con un ritmo lento y, para decirlo en los términos utilizados por Simmel, había fuertes lazos sociales (sin que esto fuera siempre positivo), así como limitaciones a la diferenciación individual. Estos contrastes les implicaron importantes retos para adaptarse a la ciudad.

Sin embargo, es preciso puntualizar los tipos de procesos migratorios que experimentaron las y los jóvenes de este estudio para comprender de mejor manera su impacto en su salud mental. Los motivos para migrar relacionados con sus expectativas de continuar sus estudios y mejorar sus formas de vida (expectativas que en gran medida resultaron satisfechas) contribuyeron a vivir la llegada a la Zona Metropolitana de Oaxaca como una experiencia deseada y valorada, en la que no todos los aspectos de la cultura que dejaban atrás dolía perderlos. Además, debe tomarse en cuenta que se trató de una migración regional del campo a la ciudad, que facilitó el retorno constante a sus comunidades de origen. Ese “ida y vuelta” físico y simbólico les permitió a las y los jóvenes mantener vínculos estrechos con sus familias y comunidades, atenuando la experiencia de duelo y desarraigo que caracteriza a las migraciones internacionales^75^. También, por su condición juvenil (principalmente el rasgo de ser solteros y sin hijos), la familia que dejaban atrás era la de origen y no la de procreación, lo que produjo una experiencia distinta a la de los migrantes adultos, quienes suelen dejar atrás a sus parejas e hijos y suelen añorar la vida familiar que dejaron en sus comunidades de origen^75^. Entre la mayoría de las y los jóvenes de este estudio su futuro imaginado se encontraba fuera de su comunidad.

Estos elementos pueden contribuir a explicar la experiencia migratoria de las personas participantes de este estudio que, si bien representó un reto para la adaptación, no significó *per se* una fuente de sufrimiento social importante. Fueron las peculiaridades del contexto de llegada las que identificaron como fuente de su sufrimiento psíquico, el cual devino en la producción de lo que identificaron como entidades psicopatológicas.

Estas peculiaridades se refirieron a una actitud citadina ambigua respecto de su condición étnica en articulación con otros ejes de discriminación como el colorismo y el clasismo, en tanto manifestación de la ideología del privilegio blanco que, como han señalado estudios previos, contribuye al establecimiento de las jerarquías raciales en nuestro país^75^.

Esta actitud se caracterizó por presentar un discurso de respeto a las diferencias a la par que se producían actitudes de violencia y discriminación en los distintos espacios donde desarrollaban sus vidas, centralmente en los espacios escolares y frente a algunas formas de atención a las que recurrieron. Cabe destacar que esta discriminación no fue necesariamente basada en un trato en el que se negara explícitamente un trato igualitario, pues las personas jóvenes que participaron en el estudio tuvieron un acceso efectivo a las instituciones educativas y sanitarias, públicas y privadas. Se trató más bien de un conjunto de prácticas que produjeron resultados desiguales afectando el acceso a ciertos derechos y reproduciendo así la desigualdad social, lo que de acuerdo con Solís^76^ puede considerarse también discriminación. Esto destaca la importancia de la comprensión de la actitud de la cultura de acogida ante los inmigrantes para la comprensión del impacto de la migración en la salud mental^16^ y reitera la importancia de que las intervenciones en salud mental en estos colectivos incluyan estrategias dirigidas a la desarticulación de estas violencias que, como se ha señalado, comprenden desde las agresiones interpersonales (lo que Scheper-Hughes llamaba violencia de todos los días) hasta formas de violencia estructural referidas a las organización política y económica de la sociedad que impone a los sujetos condiciones de opresión e inequidad^77^.

Los trastornos construidos a partir de los procesos descritos se enmarcaron en los de tipo ansioso y depresivo, abuso de sustancias e ideación suicida, los cuales se encuentran entre los problemas generalmente referidos como “menores” o “no graves” cuya prevalencia se ha incrementado durante la pandemia por covid-19 y las estrategias de contención desarrolladas^78^. Estos problemas son también los de mayor prevalencia para esta categoría etaria en la población general^79^ y en adolescentes que viven en comunidades indígenas^67^.

A diferencia de estudios que han identificado concepciones indígenas de persona en las que se niega una separación de la mente y el cuerpo^26^^,^^65^ y que han analizado síndromes de filiación cultural con manifestaciones a la vez psíquicas y orgánicas^26^^,^^29^, en este estudio se identificó una “occidentalización” en las formas de nombrar, explicar -y con ello producir- el trastorno. Esto puede deberse a que los sujetos, al integrarse al contexto urbano desempeñando roles específicos (estudiante, novio, novia, amigo, amiga) en espacios particulares (escuela, lugares de ocio y diversión) se exponen a problemática “juveniles” que producen malestares emocionales a los que nombran (y, en ese sentido, elaboran y significan) utilizando categorías nosológicas provenientes de los discursos escolares, mediáticos y sanitarios a los que han estado expuestos durante su trayectoria vital y que hacen parte de los saberes psicológico-psiquiátricos hegemónicos en el contexto urbano al que se han integrado. Esto coincide con los planteamientos de estudios previos^80^ sobre los complejos procesos de desindianización en personas indígenas que se integran a la vida urbana en Perú, que identificó que los indígenas urbanos mantienen una conciencia de otredad respecto de los no indígenas basada en diversos marcadores culturales, y que integran formas de conocimiento del contexto urbano, al que se suelen subordinar los conocimientos propios. 

Se ha señalado que las poblaciones indígenas enfrenten barreras de acceso a servicios de salud relacionadas con su residencia rural y con su condición de pobreza y marginalidad^19^^,^^24^; sin embargo, en este estudio se trabajó con un grupo de jóvenes que residen en un contexto urbano y cuya posición social no es -en la mayoría de los casos- marginal. Esto favoreció la disponibilidad y el acceso a diversas formas de atención, públicas y privadas, pero se identificó otro tipo de exclusión, que afecta ya no a las personas, sino a sus saberes. 

Estudios previos sobre trayectorias de atención de personas indígenas de enfermedades en general o de la salud mental en particular^42^^,^^43^^,^^44^^,^^51^ reportaron un uso articulado de la medicina indígena y la biomedicina a lo largo de la trayectoria. Sin embargo, en este estudio se identificó una escasa utilización de la medicina tradicional y un limitado conocimiento de sus fundamentos teóricos y simbólicos o, mejor dicho, una importante reinterpretación de estos conocimientos, en los que se aprecia su articulación con una lógica cientificista y evolucionista para explicar la producción de los trastornos mentales y su atención. Este proceso parece ir contracorriente de lo que se propone desde la salud mental colectiva: el reconocimiento de que lo mental no debería considerarse como separado de lo orgánico^81^ y una crítica y distanciamiento de la lógica cientificista.

En cuanto a las características de las trayectorias analizadas, se identificaron algunas similitudes con estudios previos realizados en Argentina, Chile y México^46^^,^^47^^,^^48^^,^^49^^,^^50^, a pesar de que estos últimos trabajaban con poblaciones diferentes (por ejemplo, adultos, niños o adolescentes infractores, en ningún caso indígenas), o con problemas de salud mental distintos (como problemas del desarrollo), se centraban solo en el consumo de sustancias como el trastorno eje, o algunos se limitaban a estudiar las trayectorias institucionales dentro del sistema de salud oficial^47^.

Entre las similitudes mencionadas se destaca la heterogeneidad de las trayectorias analizadas, tanto en su extensión en el tiempo, como respecto a la variedad de formas de atención utilizadas, tales como servicios psicológicos y psiquiátricos -públicos y privados-, profesionales (psicólogos y trabajadores sociales) de instituciones escolares y gubernamentales; y terapias grupales y holísticas. En cuanto a la valoración que hacen los sujetos de las formas de atención que utilizaron, se coincide en identificar señalamientos críticos a la escasa empatía de algunos profesionales, su trato impersonal, sus limitaciones para comprender el trastorno que aqueja a sus consultantes, la escasa utilidad de algunas de las herramientas que aportan y las relaciones asimétricas entre especialistas y consultantes (especialmente en el caso de la psiquiatría, que es la forma de atención más criticada). Por su parte, las personas consultantes valoraron de manera positiva las relaciones terapéuticas profesional-paciente horizontales y empáticas, la comunicación fluida con los psiquiatras para superar la desconfianza en la medicación, y las intervenciones colectivas (incluso las que se desarrollaban al interior de instituciones hospitalarias) que permitían conocer las vivencias de los compañeros y posibilitaban las relaciones de confianza y la comprensión entre pares. También valoraron positivamente las terapias holísticas tales como meditación, yoga, homeopatía y acupuntura. Estas opciones eran generalmente utilizadas por sectores medios y no fueron utilizadas por las y los participantes de nuestro estudio; sin embargo, estos sí valoraron algunos elementos relacionados con estas terapias que encontraban en la forma de atención colectiva más utilizada por los jóvenes varones: los grupos de ayuda mutua, como fueron una concepción integrada mente-cuerpo y la búsqueda del autoconocimiento y la transformación espiritual.

Nuestro estudio, al trabajar con jóvenes indígenas, presentó ciertas particularidades, destacando el papel central de las violencias relacionadas con el racismo y la discriminación como determinantes de la construcción de los trastornos mentales, el papel de los saberes y recursos tradicionales para la atención de los trastornos y para la autoatención en general, así como retos específicos para el acceso efectivo a las formas de atención de su interés, debido a su posición en el entrecruce de diferentes ejes de desigualdad: la raza o etnia, la clase social y en algunos casos, el género. 

En este sentido, se pudo identificar que, a pesar de que se ha documentado la presencia cada vez mayor de personas indígenas en las ciudades y la diferenciación social al interior de estos grupos^82^, persisten las dificultades para su reconocimiento en diversos ámbitos. En los servicios psicológicos se identificó una representación de la población indígena limitada (centrada en la lengua o el lugar de origen), estereotipada (en tanto pobres), racializada y anclada a las comunidades, similar a otros trabajos en los que se ha evidenciado representaciones estereotipadas del indio como campesino y analfabeto^80^, así como dificultades para definir lo indígena en fuentes de información oficiales^83^, evidenciando un patrón de invisibilización de los sujetos étnicos y de sus necesidades particulares.

Sin embargo, se plantea que esta limitación para el abordaje de la diversidad, que caracterizó el modelo de atención de la psiquiatría y los abordajes psicológicos individualistas y más cercanos a la concepción biologicista y universal de la enfermedad mental, es una manifestación de una limitación más amplia de la psiquiatría actual -y de algunos abordajes de la psicología- para la comprensión de los determinantes socioculturales de la salud mental. Este fenómeno puede interpretarse como resultado de los ecos de lo que algunos autores han denominado el “rearme neokraepeliniano” en la psiquiatría estadounidense de la década de 1970 -que a su vez produjo sus correspondientes movimientos de resistencia- caracterizado por una vuelta a la visión moderna positivista que centra su interés en la investigación biológica, el afán clasificatorio de los trastornos, la expansión de la psicofarmacología y la medicalización de la vida^84^.

Algunos autores^19^ han interpretado esta situación en términos de una inadecuación cultural, al no integrar la cosmovisión y formas de vida de los pueblos indígenas en la implementación de los servicios; sin embargo, se plantea que esta interpretación no integra un elemento fundamental, presente en los procesos analizados, que es la superioridad asumida del conocimiento occidental sobre el no occidental, por lo que se prefiere utilizar la categoría de racismo epistémico^85^, que implica una imposición de saberes que se expresa en el campo de la “salud mental” a través de lo que se ha denominado “colonialidad interna de la psicología”^86^ en tanto disciplina que legitima un sistema de representaciones adecuadas al proyecto de la modernidad^87^ y al servicio de la reproducción del capital^85^. Representaciones que impregnaron los imaginarios de los actores entrevistados y que permiten explicar por qué, a pesar de que las y los jóvenes refirieron situaciones que evidenciaban una falta de adecuación cultural, no interpretaron esa falta de adecuación como una barrera para la atención ni le dieron central importancia, evidenciando una naturalización de las exigencias sociales de adaptación a las concepciones, procesos y valores vigentes en el contexto urbano donde las y los jóvenes desarrollan sus vidas. 

Revertir estos procesos discriminatorios se ha señalado como una prioridad para la política sanitaria regional que recomienda garantizar los derechos humanos de personas con problemas de salud mental y abordar cuestiones de género, racismo y discriminación como determinantes de la salud mental^88^. Desde diferentes frentes institucionales, políticos y académicos se ha propuesto el enfoque intercultural como una vía para lograr estas metas y, siguiendo las directrices del enfoque, se han realizado acciones en la región tales como las experiencias de hospitales interculturales, el desarrollo de departamentos de psiquiatría transcultural en centros de investigación y de atención a la salud, la inclusión de síndromes culturales en el DSM-5, o las propuestas de instrumentos adaptados a la cultura para indagar aspectos de la salud mental de poblaciones indígenas^24^^,^^89^. No obstante lo promisorio de estas propuestas y acciones, será necesario atender a las posibles contradicciones entre los discursos oficiales y las prácticas^90^ y al riesgo de instrumentación del discurso intercultural al servicio de lógica de reproducción de las relaciones de dominación existentes. 

## CONCLUSIONES

Las y los jóvenes indígenas participantes de este estudio vivieron problemáticas juveniles globales, pero matizadas por sus particularidades étnicas y su contexto específico, caracterizado por una actitud ambivalente hacia lo indígena y los indígenas. Estas condiciones produjeron formas específicas de enfermar, nombrar la condición patológica y atenderla, caracterizadas por la occidentalización de sus concepciones y experiencias y por la superación de barreras de acceso a servicios de salud mental, aunque con limitaciones en cuanto a la pertinencia cultural. 

En estos procesos se evidenció una acción trasversal de lo que puede denominarse racismo estructural que jerarquiza personas y saberes y que impregna los modos de vida, las interacciones personales, las dinámicas institucionales y los saberes profesionales y populares. Un racismo que se puede manifestar también en las concepciones que romantizan la vida comunal y que no exploran su rol en la producción del malestar psíquico. 

Se propone que el papel del racismo en la construcción de los trastornos mentales y su atención excede al generalmente reconocido, centrado en las interacciones personales entre pares y entre profesionales de la salud y pacientes. Si bien esta forma de racismo perdura, es perniciosa y es preciso trabajar en su superación, es importante identificar otras expresiones de racismo, tales como la invisibilización de sujetos y colectivos y la colonialidad de los saberes. 

Consideramos importante puntualizar que el abordaje crítico que se ha realizado en los procesos de desindianización u occidentalización de las formas de vida y pensamiento de las y los jóvenes indígenas, y su relación con la construcción y atención del trastorno mental, no implica un posicionamiento esencialista que rechace el cambio cultural. Lo que se cuestiona son las condiciones de ese cambio, caracterizado por una jerarquización de saberes y modos de vida que invisibiliza a los sujetos y sus necesidades y que favorece una adecuación de los sujetos respecto a los saberes hegemónicos, en sentido inverso al que se aspira en las propuestas interculturales.

Algunas de las corrientes psicológicas y formas de autoatención mencionadas por los participantes parecieran tener rasgos teóricos o procedimentales que favorecen el abordaje de la diversidad cultural, y la desigualdad social. Se plantea la conveniencia de profundizar en su análisis para establecer su potencial para el desarrollo de abordajes decoloniales en el campo de la salud-enfermedad mental, y se propone que el fortalecimiento de los diálogos interdisciplinarios entre las disciplinas del campo psi y las ciencias sociales, entre ellas, la sociología y la antropología médica, pueden contribuir en este sentido, sobre todo si logran traspasar los límites de la academia y reflejarse en aspectos prácticos de la formación profesional y continua de los profesionales encargados de la atención a la salud mental. 

Por último, cabe señalar que entre las limitaciones del estudio se señala la dificultad que se tuvo para contactar con los sujetos de estudio (las y los jóvenes), lo cual pudo afectar la variabilidad de la muestra. Esta situación se atribuye a que la identificación como indígena y como persona con un trastorno mental supone un doble estigma, lo que limita la visibilización dentro de los colectivos de los sujetos con estas características. Otra limitación fue que, por el tiempo disponible acotado, no se realizaron entrevistas con profesionales que trabajaran en instituciones públicas de seguridad social o para la atención a población no derechohabiente (por el tiempo que suelen tomar los trámites para la autorización del trabajo de campo al interior de estas instituciones). También por limitaciones de tiempo no se profundizó en la recopilación de datos sobre una forma de atención que emergió durante las entrevistas: la religiosa. Por consiguiente, se recomienda seguir desarrollando líneas de investigación que integren estos espacios y actores, que profundicen en líneas que en este estudio son solo esbozadas -tales como las implicaciones del género en la conformación de las trayectorias- y que aborden el tema en otros tipos de poblaciones en términos étnicos, etarios, de residencia y de clase social, a fin de comprender las diferentes aristas de los procesos estudiados. También se reconoce como una limitación la dificultad, generalmente presente en los estudios de corte biográfico, para contrastar los relatos de las y los participantes con otro tipo de datos, a fin de lograr una mejor aproximación a la manera en que se desarrollaron las trayectorias. 

## MATERIAL SUPLEMENTARIO

Sistematización de las trayectorias de atención analizadas (ver Material suplementario).
