# Supplementary material for: Racismos y salud mental en jóvenes indígenas residentes en la Zona Metropolitana de Oaxaca, México
Source: Salud Colect. 2024 Dec 2;20:e4908. [Article in Spanish] doi: 10.18294/sc.2024.4908 (PMC11925741; doi:10.18294/sc.2024.4908)
Supplement: Translation

# Translation: Racism and mental health among indigenous youth living in the Metropolitan Area of Oaxaca, Mexico

**Authors:** María Alejandra Sánchez Bandala, Paola María Sesia

**Affiliations:** 1 PhD in Anthropology. Professor-researcher, Universidad de la Sierra Sur; Miahuatlán de Porfirio Díaz, Mexico. alejandra.bandala1@gmail.com; 2 PhD in Anthropology. Professor-Researcher, Center for Research and Higher Studies in Social Anthropology-South Pacific; Oaxaca de Juarez, Mexico. sesia@ciesas.edu.mx

**Keywords:** Indigenous Peoples, Mental Health in Ethnic Groups, Mental Health Services, Racism, Mexico

## Abstract

Indigenous populations show high prevalence rates of mental disorders and limited access to mental health services. The aim of this study was to analyze the mental health care trajectories of indigenous youth living in the Metropolitan Area of Oaxaca, Mexico. Between May and August 2023, we conducted a qualitative study involving non-participant observation, in-depth interviews with seven indigenous youth, and semi-structured interviews with nine health professionals, healers, or leaders of mutual support groups. We identified Westernization processes, not exempt of tensions, in how these young people perceive themselves as both young and indigenous, in the development of their mental disorders, and in the ways they seek treatment. Their care involved the use of psychological and psychiatric services, mutual support groups, and, to a limited extent, traditional medicine. This study concludes that these dynamics are interwoven and amplified by interpersonal, institutional and epistemic racism, which must be dismantled to improve the mental health of indigenous youth.

## INTRODUCTION

The growing burden of mental disorders, coupled with treatment gaps, represents one of the central public health challenges in the Americas.^1^ Despite widespread acknowledgment of the urgency of this issue, there is no single way to conceptualize mental health and illness. Some perspectives adopt the medical model, emphasizing a biologicist and individualistic approach focused on psychopathology.^2^ Moving beyond this reductionist view, although still trapped within an individualist framework, the World Health Organization (WHO) defines mental health as “a state of well-being in which the individual realizes their abilities, copes with the normal stresses of life, works productively, and contributes to their community”.^3^^)^ In contrast, the Latin American collective health framework understands illness - including mental illness - as a multifaceted marker linked to biological, cultural, historical, and social dimensions. This perspective aims to comprehend the “collective fabric in which illnesses are generated and addressed”, opening possibilities for developing a “comprehensive therapeutic approach”.^4^ Within this framework, collective mental health focuses on the study of “experiences of affliction”, recovering the “social world” of psychological suffering.^5^ It addresses the social determinations of mental health, particularly the violence embedded in material living conditions and power dynamics. At the same time, it acknowledges the agency of individuals experiencing psychological suffering, valuing their understanding of their processes and their participation in health care strategies.^6^

As one can observe, mental health and health can be conceptualized in various ways, often reflecting opposing perspectives. For example, some define mental health as the individual capacity to adapt, cope with the “normal” stresses of life and adversity, and maintain harmony in relationships.^3^ In contrast, others view it as the ability to transform living conditions.^7^

This polysemy also extends to how we describe what might be considered the opposite of mental health. The concept of mental illness typically refers to discrete entities aligned with biomedical approaches. However, this term has become less common in academic literature, where “mental disorder” is now preferred. According to Braunstein, ”mental disorder” remains a euphemism for a construct that closely resembles mental illness.^8^^)^ The WHO defines a mental disorder as “a clinically significant disturbance in an individual’s cognition, emotional regulation, or behavior”.^9^ Definitions of mental disorders often incorporate criteria such as deviation from norms, functional limitations, experiences of distress, and a notion of dangerousness (though the latter two criteria have been subject to debate).^10^ In practice, mental disorders refer to constructs that align with the diagnostic criteria outlined in dominant nosological classifications, such as the *International Classification of Diseases* (ICD) and the *Diagnostic and Statistical Manual of Mental Disorders* (DSM).^11^

Critical approaches employ alternative categories to describe experiences distinct from mental health. One such category is *psychic suffering*, understood as a harmful experience perceived as subjective but rooted in the network of relationships surrounding the individual.^12^ These forms of suffering are seen as part of life’s challenges that, while causing distress, are not pathological. This concept aligns somewhat with the idea of *malaise* although some authors use the latter to emphasize the social conflicts and contradictions (often not consciously recognized by individuals) that underlie such experiences.^13^^)^ In this study, we use the terms *illness* and *disorder* to explore the constructs participants perceive as pathological, while we apply the categories of *suffering* or *discontent* to experiences of distress not necessarily regarded as pathological by the individuals themselves.

When addressing the mental health of Indigenous populations, two major perspectives emerge. Epidemiological studies and critical medical anthropology - focused on power asymmetries and structural inequalities^14^^,^^15^^)^ - have identified globally high prevalence rates of disorders such as depression, substance abuse, alcoholism, and suicide among Indigenous populations, often exceeding those of non-Indigenous counterparts. These conditions are linked to social stressors stemming from migration,^16^ discrimination, loss of culture, and diminished control over living conditions.^17^^)^ For instance, a study in Mexico that analyzed 2014 official mortality data reported that the national suicide rate among Indigenous men (including adolescents and young adults) was higher than among the general population. Additionally, the mortality rate related to alcohol use was 2.5 times higher among Indigenous compared to non-Indigenous peoples.^18^ In Colombia, another study found that the suicide rate among Indigenous populations reached over 500 per 100,000 inhabitants, more than 100 times higher than the national average of 4.4 per 100,000 inhabitants.^19^ Indigenous peoples also face worse indicators for the availability of and access to biomedical health services, primarily due to rural residency and poverty.^20^^,^^21^ Moreover, the care they receive in biomedical services is often inadequate. Biomedical professionals tend to overlook cultural syndromes, reframe their suffering within Western psychiatric categories, neglect the socio-relational aspects of their conditions, and ignore or reject their community-based therapeutic resources.^19^^,^^22^^,^^23^ This occurs despite an extensive legal and institutional framework at both national and international levels promoting intercultural and rights-based approaches to Indigenous health care.^24^^,^^25^

In contrast, approaches that acknowledge the cultural relativity of defining and experiencing mental health and illness emphasize the need to understand these dynamics through the worldviews of Indigenous peoples, rather than forcing them into Westernized frameworks. This perspective has revealed that some Indigenous communities lack an equivalent concept for “mental health,” and when such a concept exists, it differs from the Western biomedical notion, focusing instead on elements like spirituality, well-being (*buen vivir*), and/or harmony with the community and nature.^17^

Fields such as medical anthropology, ethnopsychiatry, and transcultural psychiatry have produced extensive literature aimed at understanding “culturally bound syndromes” like *mal de ojo* (evil eye), *aire* (air), *nervios* (nerves), *susto* (fright), *envidia* (envy), *vergüenza* (shame), *muina* (anger), or *bilis* (bile). These conditions are characterized by both physical and psychological manifestations, and various traditional therapeutic practices addressing them have been documented.^23^^,^^26^^,^^27^^,^^28^^,^^29^ However, critiques have pointed out that some studies focus exclusively on traditional medicine without delving into its real-world interactions with modern medicine, which ultimately limit their explanatory scope.^30^

This highlights the need to approach Indigenous health-illness-care processes by acknowledging the significance of cultural change, which has intensified in recent decades due to factors like migration, increased levels of education, and access to various information and communication technologies.^31^ These conditions position young, Indigenous, and migrant individuals as key subjects for exploring processes of cultural transformation and their impact on how they conceptualize mental health and illness, as well as how they prevent and address related challenges, likely drawing from multiple forms of mental health care.

A useful theoretical and methodological tool for addressing the complexity of these dynamics is the concept of *care trajectories*, understood as the steps individuals take to seek therapy in response to a health issue, often involving different therapeutic options^32^. Osorio suggests that this approach analyzes the decisions and strategies individuals use to confront-and, in the process, make sense of-their episodes of suffering.^33^

The concept of *care trajectories* has been widely used to study various health-illness-care processes. ^34^^,^^35^^,^^36^^,^^37^^,^^38^^,^^39^^,^^40^^,^^41^ These studies have highlighted the sequential or parallel use of different therapeutic options and the significance of self-care dynamics, and have provided insight into the meanings and subjective experiences of patients and their families. Some research has focused on the care-seeking behaviors of Indigenous populations or those living in rural settings, revealing the syncretism and complementarity between biomedical and traditional knowledge systems.^42^^,^^43^^,^^44^^,^^45^ However, studies on care trajectories in mental health remain scarce in the global literature^46^^,^^47^^,^^48^^,^^49^ or in Mexico,^50^ and are even rarer when focusing on Indigenous populations.^51^

This article stems from a broader research project titled *Care trajectories in mental health among indigenous youth living in the Metropolitan Area of Oaxaca*. In Oaxaca, the epidemiological burden of several mental disorders is on the rise. Among young people, certain disorders are particularly prevalent: for example, the incidence of depression in women rose from 47 to 77 per 100,000 inhabitants between 2014 and 2022,^52^ while suicides are most common among individuals aged 15 to 29, particularly men.^53^ The state of Oaxaca has the highest percentage of Indigenous language speakers in the country (31.2%, compared to the national average of 6.1%), with 69% of the population self-identifying as Indigenous,^54^ and experiences significant rural-to-urban migration.^55^^)^

The Metropolitan Area of Oaxaca comprises the city of Oaxaca de Juárez, the state capital with the largest Indigenous population in the region, and 22 surrounding municipalities. In terms of public mental health services, the state has a single psychiatric hospital, “Cruz del Sur,” located in the southern suburbs of Oaxaca City, and seven Specialized Units in Addiction Care (UNEME-CAPA for its acronym in Spanish), two of which are in the metropolitan area.^56^ Additionally, there are two Youth Integration Centers, one located in the Metropolitan Area of Oaxaca. For individuals covered by the Mexican Social Security Institute (IMSS), this institution provides psychological consultations at three first-level Family Medicine Units in the Metropolitan Area of Oaxaca and psychiatric consultations at the General Hospital in Oaxaca City.^57^ The largest support network, however, comes from mutual aid groups: in Oaxaca City and its metropolitan area, there are 81 Alcoholics Anonymous centers^58^^)^ and 18 Neurotics Anonymous centers.^59^

This geographic and sociocultural space, is a hub for internal migrants - primarily from rural areas of the region - and for international migrants in transit. Along with its self-proclaimed multicultural identity, migration presents a compelling case for exploring the research topic. This article focuses on analyzing how ethnic identity and the sociocultural structure in which it is embedded and constructed shape specific ways of experiencing and addressing mental health issues within the framework of the care trajectories we could study.

## METHODOLOGY

A qualitative approach guided the study. Participants included Indigenous youth aged 18 to 30, residing in the Metropolitan Area of Oaxaca, who reported seeking care for a “mental health” issue, as well as health professionals, healers, and leaders of mutual aid groups providing mental health services in the study area. Notably, four of these professionals had a therapeutic relationship with the interviewed youth. We also conducted non-participant observation in the spaces where we requested collaboration, to facilitate contact with the interviewees. Fieldwork took place from early May to late August 2023.

We conducted in-depth interviews with the youth participants (four women and three men) to explore their health care trajectories. We carried out semi-structured interviews with health professionals (five participants), healers (two participants), and mutual aid group leaders (two participants). These interviews focused on understanding the main characteristics of the care offered to young people - both Indigenous and non-Indigenous - experiencing mental health disorders. Tables 1 and 2 provide detailed characteristics of the participants.

**Table 1:** General characteristics of the indigenous youth participants. Metropolitan Area of Oaxaca, Mexico, 2023.

Pseudonym	Age	Marital Status	Place of Birth	Migration Experience	Places of Residence	Indigenous Language	Religion	Level of Education	Occupation	Social Security Coverage
Alma	22	Single	Ciudad Juárez Chihuahua	At the age of 16, she migrated to the MAO to escape school bullying in her place of origin	Oaxaca de Juárez (MAO)	None	She identifies as Catholic but hardly practices it.	Undergraduate student	Student	None
Julia	29	Married	Santa Catarina Juquila	At the age of 15, she migrated to the MAO to study high school	San Bartolo Coyotepec (MAO)/Miahuatlán de Porfirio Díaz	Chatino	Catholic	Bachelor's degree in Nursing	Nurse	ISSSTE
Paula	28	Common-law marriage	Asunción Cacalotepec	At the age of 13, he migrated to the MAO to study secondary school.	San Sebastián Tutla (MAO)/ Miahuatlán de Porfirio Díaz	Ayuuk (Mixe)	Catholic	Bachelor's degree in Nursing	Postgraduate student	IMSS-Bienestar
Magali	29	Single	Cuilapam de Guerrero (MAO)	None (was born and currently lives in the MAO).	Cuilapam de Guerrero (ZMO)	None	None	Bachelor's degree in Archaeology	Archaeologist	None
Fernando	24	Single	San Juan Quiahije, Juquila	At the age of 12, they migrated to the MAO to study secondary school.	Oaxaca de Juárez (ZMO)	Chatino	Catholic	Engineering graduate	Industrial machine maintenance technician	IMSS-Bienestar
Ismael	29	Common-law marriage	Santiago Atitlán	At the age of 27, they migrated to the MAO in search of care at the Casa Hogar of Neurotics Anonymous.	Oaxaca de Juárez (ZMO) / Santiago Atitlán	Ayuuk (Mixe)	Catholic	High school incomplete	Musician	It seems to be IMSS-Bienestar (he wasn't sure)
Nicolás	26	Single	Miahuatlán de Porfirio Díaz	Migrated to the MAO at the age of 5, along with his family, who was seeking better job opportunities.	Santa María Atzompa (MAO)	rudimentary Zapotec	None	High school and unfinished college degree	Mekanic	None

Source: Own elaboration. MAO = Metropolitan Area of Oaxaca. ISSSTE = Institute of Security and Social Services for State Workers. IMSS-Bienestar = Subsidiary of the Mexican Institute of Social Security serving the population without social security.

**Table 2:** General characteristics of the participants (professionals, healers, and leaders of mutual support groups). Metropolitan Area of Oaxaca, Mexico, 2023.

Participant	Gender	Age	Indigenous language	Education
Healer and midwife	Woman	84	Zapotec	Empiric
Healer	Woman	60	Zapotec	Empiric, diploma in Herbal Medicine
Private practice psychologist	Woman	30	Understands some Mixe, but does not speak it.	Bachelor's Degree in Psychology
Public service psychologist 1	Woman	-	None	Bachelor's Degree in Clinical Psychology, Specialist in Couple and Family Therapy
Public service psychologist 2	Woman	32	None	Bachelor's Degree in Psychology
Public service psychologist	Man	30	None	Bachelor's Degree in Psychology, Master's Candidate in Humanistic Psychotherapy
Private practice psychiatrist	Woman	30	Understands very little Zapotec.	Surgeon, specialist in Psychiatry
Alcoholics Anonymous group representative	Man	48	Understands very little Zapotec.	Bachelor's Degree (Not Health-Related)
Narcotics Anonymous group representative*	Woman	37	Mazatec	Bachelor's Degree (Not Health-Related)

Source: Own elaboration. * She also shared her trajectory of care during her youth, information that was taken for the analysis due to the richness of the information.

We began to develop the interview guides and analysis plan deductively, deriving themes from the research objectives and the state of the art, while remaining open to emergent themes. The predefined thematic lines focused on criteria for self-identifying as Indigenous youth, conceptions of mental health and illness/disorder, the impact of living conditions on health and illness/disorder, representations of the ascribed disorder, and health care trajectories. We used these thematic lines as analytical categories, to create a set of initial codes.

We present the results according to these thematic lines, described in Table 3, which outlines the correspondence between subtitles, themes, and codes. Italicized codes in the table emerged during data analysis and were integrated inductively. These emergent codes did not introduce entirely new themes but revealed unexpected ways participants conceptualized the processes under study.

**Table 3:** Correspondence between subtitles, thematic lines, and codes.

Subtitle	Thematic Lines	Codes
Indigenous Youth: Tensions in the Construction of Identity	Criteria for Identifying as an Indigenous Youth	Criteria for Youth
		Indigenous Criteria
The Community Environment, Experienced Violence, and the Production of Emotional Distress	Living Conditions and Their Impact on Health-Illness/Mental Disorder	Determinants of Well-being
Urban Life and Mental Health: Between Freedom, Anonymity, and Discrimination	Living Conditions and Their Impact on Health-Illness/Mental Disorder	Determinants of Psychological Suffering
Characterization of Distress and Assumed Diagnoses	Conceptions of Health and Illness/Mental Disorder	Conceptions of Health-Mental Illness
	Representations of the Ascribed Disorder	Ascribed Diagnosis and Manifestations
		Ascribed Etiology
		Ascribed Prognosis
Forms of Care Used and Their Cultural Relevance	Care Trajectories	Identification of Abnormality
		Initiation of Halth-Seeking
		Traditional Medicine*: Reasons for Seeking Care, Availability and Access, Diagnosis, Treatment, Adherence and Discontinuation, Outcomes, Experience, Ethnic Specificity and Appropriateness

Source: Own elaboration. * Codes similar to the last eight were constructed and applied to each of the forms of care that the study subjects used: traditional medicine, biomedical care, psychological care, psychiatric care and mutual aid groups.

For example, while the initial plan included examining the impact of living conditions on health and mental illness/disorders, the data suggested the need for a code addressing experiences of distress that participants did not perceive as pathological. This led to the creation of the code *determinants of psychological suffering*. Similarly, the initial plan included a code for participants’ diagnoses. However, as many participants self-diagnosed themselves rather than receiving a professional diagnosis, we renamed this code *ascribed diagnosis and manifestations*.

It is important to note that these changes were not limited to the code names but extended to the scope and referents of each code, encompassing the “real” or potential content they addressed. 

We recorded the interviews with participants’ consent, obtained through signed informed consent forms, and transcribed for subsequent coding and systematization using Atlas.ti software, version 8. To ensure confidentiality and anonymity, we assigned pseudonyms to all participants.

Non-participant observation took place in the spaces where we sought collaboration to establish contact with potential interviewees. The observation focused on documenting how institutional spaces represented Indigenous youth, their mental health, and appropriate ways to address their mental health needs. We applied a thematic content analysis to the data derived from both interviews and observations.

The project received ethical approval from the Health Research Ethics Subcommittee of the Center for Research and Advanced Studies in Social Anthropology, internal file number 005.

## RESULTS

### Indigenous youth: Tensions in identity construction

Most of the youth interviewed had completed higher education and reported ongoing mobility between the Metropolitan Area of Oaxaca and their places of origin (typically in rural or semi-urban contexts). This mobility has also led to sociocultural shifts, creating tensions in how they conceive of themselves as “youth.” While in the Metropolitan Area of Oaxaca the category of youth is fully legitimized as part of the life cycle, in their places of origin it is a more recent category, emerging in relation to the availability of higher education, access to the internet, and migration dynamics.

The youth interviewed have resolved these tensions by prioritizing and internalizing a Westernized conception of youth, adopting most of the traits associated with it, such as being unmarried, playing the role of a student, assuming a degree of freedom and autonomy from their parents, and claiming the right to socializing and having fun with peers. However, this identity is questioned by their relatives who remain in their communities of origin. These relatives often communicate the social expectation for them to transition into adulthood at ages that, in urban contexts, align with adolescence (around 13 or 14 years old). Although none of the participants reported experiencing significant emotional distress due to these situations, they did express some confusion and frustration at not being able to fully adapt their lifestyles, practices, and subjective experiences to fit the stages considered universal in the Western life cycle framework.

Regarding their ethnic self-identification, the young people interviewed identified a range of traits that distinguished them from “mestizo” people, primarily language and customs and traditions, although some also included phenotypic elements like skin color. However, they expressed a preference for being identified by the demonym of their region or town, or by the name of their specific ethnic group/language (Zapotec, Mixtec, Chatino, etc.), rather than the generic term “indigenous.” One of the young people reflected on this preference, linking it to experiences in the urban context where the term “indigenous” was often used to convey mockery and disdain.

“… *maybe in my past that word was misunderstood, because in reality it’s not a bad word, I don’t consider it bad now, but yes, when they called me indigenous, Chatino, or Indian in high school. I don’t know how to express it, I think in my head that someone with more authority wants to belittle me when they say that* [but] *I know it’s not a bad word, it’s a source of pride to be a person with Indigenous roots*.” (Fernando)

They reported that such experiences caused them emotional suffering; however, it is important to highlight that they also faced situations in their communities of origin that generated various degrees of discomfort, as described in the following section. Notably, they considered that some of these situations originated their mental disorders.

### The community environment, the violence experienced, and the production of emotional distress

The people interviewed described a great variety of emotional distress, although this variety shared a common denominator: it was related to different types of violence they experienced in their communities of origin. Ismael, one of the young people interviewed, mentioned that the violence he witnessed from his father toward his mother, especially when his father became intoxicated, contributed to the development of his “neurosis.” Similarly, Nicolás said that his father’s alcoholism and the violence he inflicted on his mother had emotionally affected him. While domestic violence is not exclusive to Indigenous contexts, the narratives analyzed revealed how the permissiveness of the environment toward male control over women’s lives, or the existence of arranged and often forced marriages - characteristic traits in some rural and Indigenous communities in Mexico - contributed to this type of violence.

Alma, on the other hand, pointed out that the *envy* her classmates felt toward her for excelling academically contributed to the bullying she experienced, which she identified as one of the triggers for her depression. She noted that such dynamics, based on envy, are part of the cultural traits of her community of origin. In her case, it was also evident how community values about the importance of family unity and loyalty allowed for the impunity of sexual violence perpetrated by relatives.

*“I think the idea of family in my town makes these things get covered up more. Because even if your family knows what happened to you, but it was… I don’t know, your grandfather or your uncle, it’s more important for the family to maintain the bond, so it’s ‘don’t say anything because it’s your relative.”* (Alma)

Some of the professionals interviewed highlighted the importance of media in shaping expectations about family relationships and ways of being young, which can cause emotional distress for those who are unable to adopt the lifestyles and ways of relating that are legitimized by these media. One of the young people interviewed noted that comparing the image of family he saw in the media with his own family reality led to frustration, which he identified as the beginning of a series of dissatisfactions that eventually contributed to his problematic substance use.

*“…although I lived in the village, many times I didn’t accept it, because of the idea they sell you through television about what a perfect family is like* […] *I have a very clear image in my mind of when I used to watch TV, and I’d see how a family would play on the grass with their kids, with a new toy, or I don’t know, and I imagined that was a happy family, a peaceful family, but in my house, all there was, was the need to work very hard* [so] *I started to reject it, I started to want more things. So, because of dissatisfaction, I attribute that those things* [substance use] *happened.”* (Fernando)

As one can see, some of the cultural traits of the young people’s communities of origin facilitated the production of various forms of violence-whether domestic, sexual, school-related, or symbolic-with a clear gender component. These were experiences that the young people either witnessed or were victims of, and they caused them significant emotional distress.

### The urban life and mental health: between freedom, anonymity, and discrimination

Only one of the young women interviewed (Magali) was born in the Metropolitan Area of Oaxaca; the rest were originally from rural areas and had different migratory experiences. One of the young men (Nicolás) arrived as a child, as part of a family seeking better job opportunities. Three of the interviewees (Julia, Paula, and Fernando) migrated during their school years (secondary or high school), motivated by the desire to continue their studies in the city. In Fernando’s case, the motivation also included learning Spanish and improving his living conditions.

*“I decided to come, I insisted to my parents, I told them that I wanted to come study here, mostly to change, I didn’t like being in the village much, my job was to work in the field, gather firewood, help my parents with construction work, so I didn’t like it much and I decided that I wanted to learn Spanish, the goal was to learn Spanish and also finish a degree* […] *to learn more.”* (Fernando)

One of the participants migrated during her school years to escape a situation of bullying (Alma), and another (Ismael) arrived seeking help for his distress.

In some cases, the migration process included personal or family expectations that, in general, young people were able to achieve. Except for the young man who migrated seeking care at a voluntary residential center managed by the Neuróticos Anónimos movement, the rest gained access to educational opportunities and either entered or completed higher education. Moreover, the young people interviewed associated urban life with positive elements, such as the opportunity to explore new places and meet new people, going out for walks, dining out, having access to recreational spaces, and enjoying more freedom than in their communities. One of the young men also referred to a shift in mindset, noting a contrast between the mentality of those who live in rural areas and those in the city, and expressed a positive view of the urban mindset.

*“…when you’re from the village, your mind is closed, I really say this and I understand my parents, my cousins, and other people who haven’t experienced coming here or being with people who have more open, or more liberal, thoughts.”* (Fernando)

However, moving to the city also meant adapting to an unfamiliar and, at times, intimidating environment. In practical terms, as part of their daily challenges, they mentioned learning how to navigate the city or use public transportation without getting lost, or learning the local interaction codes. They also noted difficulties adjusting to the ways of relating to others, pointing out a significant difference in the type of sociability in the city compared to their hometowns. One of the young people mentioned that in the city:

*“…it’s like everyone is looking for their own thing... and there* [in my village] *it’s more like... there’s more unity, we go to bring the rain... so it will rain, and the plants will grow, the corn will grow.”* (Fernando)

Also, in relation to social relationships, some participants shared the feeling of anonymity in the city, which contrasted with the mutual recognition in their communities.

The attitude of city dwellers toward the indigenous/rural (a binomial that, in the imagination of the interviewees, seems closely linked) shaped their experience in the city. Oaxaca de Juárez (the state capital) presents itself as a multicultural city, with its main festival and tourist attractions celebrating and exalting ethnic diversity. However, we identified an ambiguous attitude toward indigeneity, characterized by both admiration and appreciation for certain elements associated with indigenous cultures, alongside contempt and rejection toward some of the bearers of those cultures, especially when these elements intersect (either in reality or in the imagination) with conditions of poverty and marginality. One of the professionals interviewed explained these contradictions as follows:

*“It depends on the season, because during the Guelaguetza month they accept* [Indigenous people] *a lot, but in other seasons, the Indigenous issue is seen as a form of discrimination. They discriminate against you for coming from a community, because you don’t wear shoes, because maybe you’re not well-dressed, because you don’t wear normal clothes, but instead wear the clothes from your region. In July, it’s well-regarded, so people take pictures, but it’s more like... I, the privileged person, am having the opportunity to be with you, I am a better person because I am spending time with you.”* (Psychologist, public service)

This ambiguity also expressed itself in the school environment, where the young people experienced both recognition and appreciation of their cultural traits (mainly their language), alongside rejection and mockery due to these and other markers, such as skin color or the condition of being a “country person,” which caused emotional distress and led them to try to hide or conceal these markers, for example, by avoiding speaking their language in public.

“*They found out* [my high school classmates] *that I spoke an Indigenous language* [...] *I still remember the word she said to me, she called me ‘Indian’* [...] *and in high school, once I started using social media, I remember that on one social media platform, an anonymous account posted something like ‘go back to your hill with your prickly pears and your donkeys.’ The comments were very derogatory*.” (Paula)

It is important to highlight that in the urban context, interviewees also encountered kind and meaningful relationships that helped them integrate into city life and provided significant emotional support. However, they also experienced repeated and hurtful situations of bullying, in a context of power imbalances between perpetrators and victims; experiences which caused psychological suffering that they generally endured in silence, without communicating their occurrence to teachers or family members. One of the young people shared that they never spoke to anyone about the bullying they endured because they thought their teachers would pity them, and their family would feel hurt, if they shared why they were harassed:

*“... it hurt when they said that to you, it’s like a deep wound, and so if I asked my brother: ‘What do you think about this?’ I think it was also a deep wound* [for him].*”* (Fernando)

The young people referred to situations that, while not directly related to their ethnic condition, could be identified as being influenced by ethnicity. For example, although school stress is a condition that can affect individuals regardless of ethnicity or residence, some of the participants mentioned that they perceived a disadvantage in their academic preparation compared to their peers who had attended previous school years in urban settings. Similarly, we found that it was common among these young people the search for a sense of belonging, a troubled quest that could encourage the initiation of substance abuse, as Fernando expressed: “The first time I smoked marijuana, I did it to be on the same level as the other person I was hanging out with, to feel good, to feel like I belonged.”

Some of the young people we interviewed mentioned that the rejection they experienced because of their ethnicity in some cases, heightened their need for recognition and acceptance. Additionally, the legitimization of heavy alcohol consumption in their communities contributed to their initiation into this practice. However, they did not necessarily perceive the relationship between experiences of rejection, what they referred to as depression, and substance abuse as unidirectional or deterministic. In some cases, what they identified as depression occurred before or alongside substance abuse, or they used substances without feeling depressed; some even saw substance abuse as a form of self-medication.

*“The first time I used it, it was a very pleasant sensation, a sensation of laughter, extreme happiness, an immense happiness, and every time I used it, I sought to return to that level of happiness* [but] *what I can also detect is that I used it for everything and for nothing, because I was sad, because I was angry, because I was anxious, because I was hungry, because I wasn’t hungry, for everything, because Mexico won, because Mexico lost.”* (Fernando)

### Characterization of distress and assumed diagnoses

At the time of the interviews, only one of the participants used an ethnomedical category from traditional medicine to name her condition (although she also used the psychological category of “trauma”). The rest used categories from psychology and psychiatry to label and interpret their conditions (Table 4).

**Table 4:** Diagnosis attribution by the study participants. Metropolitan Area of Oaxaca, Mexico, 2023.

Pseudonym	Diagnosed condition	Type of diagnosis
Julia	Trauma/fright	Self-diagnosis
Paula	Conversion disorder/anxiety	Psychological
Magali	Anxiety	Self-diagnosis
Alma	Generalized anxiety disorder with panic attacks	Psychiatric
Nicolás	Alcoholism	Self-diagnosis
Fernando	Depression and alcoholism	Self-diagnosis
Ismael	Neurosis	Self-diagnosis

Source: Own elaboration based on the data collected.

According to the interviewees, the multiple distressful experiences they lived through produced emotional and behavioral conditions that they I identified as pathological at some point later. The distress or suffering reported by each participant is described in the charts included in the Supplementary material. Sadness, depression, crying, feelings of loneliness, irrational fears, aggression, anxiety and its somatization, panic attacks, substance abuse, and suicide attempts were among the symptoms and problems identified. Throughout the trajectories analyzed, the young people named and represented their condition in different ways.

The fact that only two of the interviewees reported a diagnosis from psychology or psychiatry at the time of the interview did not mean that the rest of the young people did not seek such services at some point during their health-seeking trajectories. In fact, only one person did not seek psychological care from a licensed professional and only spoke with a psychology student who was doing her internship. We can explain this situation through other factors. On the one hand according to the narratives of the interviewees, some psychological approaches question the importance of establishing a diagnosis and prefer to work on problems without labeling their clients. On the other, two of the young people who did access psychological care mentioned that it was neither meaningful nor helpful because the psychologist did not understand their problem, or because they were reluctant to share their intimate life with her.

*“I think I didn’t go [to a second session with the psychologist] because I felt threatened that someone would know what my life had been like and the problems I was facing at that moment. I didn’t want anyone to really see who I was, what was happening in my life.”* (Nicolás)

Among the interviewees, it is notable that women preferred psychological care, which aligns with the observation of one of the psychologists interviewed, who noted that most of her clients are women. In contrast, the three men expressed a preference for mutual aid groups as a meaningful form of care. Since professional therapists do not participate in these spaces, the men attributed their condition to alcoholism, depression, and neurosis as self-diagnoses.

### Selected forms of care and their “cultural appropriateness”

The care trajectories followed by the interviewees showed great diversity in terms of duration, selected forms of care, the order in which they were utilized, experiences during care, and their perceived effectiveness.

As mentioned previously, a care trajectory is considered to begin when individuals identify an abnormal condition. In most of these cases, the identification of symptoms occurred retrospectively, once these people had internalized the etiological models proposed by the therapists they had sought and that had been meaningful to them during their trajectory. Upon identifying the abnormality, the first practices that the young people engaged in aligned with what Menéndez refers to as the self-care model.^61^

#### Self-care practices

In most cases, the young people reported that they had followed multiple self-care trajectories; self-care is understood here as the knowledge that individuals and social groups adopt and use from other forms of care, generally to ensure their biosocial reproduction and more specifically:

“*...to diagnose, explain, treat, control, relieve, endure, cure, solve, or prevent the conditions that affect their health in real or imagined terms, without the central, direct or intentional intervention of health professionals, even though they may be the reference for the self-care activity.*”^61^

It is important to note that interviewees did not limit these practices to the moments before they searched for professional help for the first time, but were continued throughout the entire health-seeking trajectory, sometimes as the only form of care at a given moment, or alongside other forms of care. Some of these practices were developed intuitively (such as taking a bath to calm anxiety), while others were based on information found on the internet; for example, meditation and mindfulness exercises. Some practices came from recommendations of previously consulted professionals.

Some of these practices were collective in nature, such as participation in support groups on Facebook, or family actions like practical help with daily tasks, advice, or emotional support. It is worth noting that these latter practices were more common among women, while among the men, the most notable form of self-care was participation in mutual aid groups, which will be discussed further later. Various forms of self-medication were also prominent, related to the consumption - not necessarily considered problematic - of tobacco, alcohol, and other substances, as a way to alleviate distress.

Two individuals recognized and valued ritual practices performed within their families as a self-care resource. Although these rituals did not specifically pursue curing or preventing any particular ailment, they were aimed at promoting health (along with other valued goods) or as a spiritual experience. Fernando, for example, participated with his family in rituals honoring Gods and Mother Earth, finding similarities between these practices, the teachings of the Bible and the Alcoholics Anonymous program. Paula, on the other hand, mentioned that in her family’s rituals, elements from “the ancestors” and Catholicism were combined, recognizing a symbolic efficacy derived from a personal decision about what to believe, as an act of faith.

“*When I went through the abortion threat, there was a time when anxiety wanted to take over again, overthinking everything, imagining the worst possible scenarios, I went through that, but at that time my mom started doing these rituals of going to the houses of the shamans, so even though I wasn’t there, in a way it comforted me to know that she was doing it* […] *not so much for medical efficacy, but because as a person, realizing that believing in something, having faith in something, can help you* […] *I think it’s not so much for medical efficacy, but more of a personal matter* […] *in this case, because I’ve focused more of my faith on the indigenous rituals we have in the community, it has helped me a lot, personally it gives me a lot of strength, but it’s a decision I decided to make.*” (Paula)

The search for professional help began in response to a variety of circumstances, including obvious physical discomfort, an inability to carry out daily activities, or emotional suffering that became unbearable. According to the participants’ accounts, this search for help was influenced by several factors, including the availability of therapeutic resources in their environment, their personal and family financial resources, previous positive or negative experiences with specific therapeutic options, the impact of the disorder on their lives, and the illness explanatory models that the individuals and/or their parents subscribed to at different points in their trajectories. The following sections describe the different forms of care used by the young people. The order in which we present them does not correspond to a sequential use throughout the trajectories, as it is impossible to identify clear chronological patterns in the heterogeneous paths followed by the participants.

### The use of traditional medicine

Young people resorted to traditional medicine primarily when they lived in their rural communities. Its use was related to its availability and accessibility in terms of proximity, cost, and consultation hours, as well as the trust they or their families had in its effectiveness. When they lived in urban areas, the use of traditional medicine was limited, despite its availability.

Susto (fright) was the most mentioned ethno-medical nosological category, and several participants acknowledged the possibility of having experienced its symptoms. Two young people mentioned visiting a healer to treat a *susto* primarily caused by an accident, with positive therapeutic results.

The healers interviewed explained *susto* as the result of the soul’s separation from the physical body, and they described the objective of the therapeutic ritual to return the soul to its body. This contrasts to the young people who sought traditional care for *susto* who emphasized the healing procedures, and only when explicitly asked did they refer to the beliefs guiding these practices. Regarding the therapeutic effectiveness of the *limpias* (spiritual cleansings) used to treat their condition of *susto*, Fernando stated that it was effective, while Julia acknowledged the possibility of its healing efficacy based on its compatibility with scientific knowledge.

“*There are certain things that I believe do help, for example, some medicinal plants are very beneficial because, in reality, pharmaceuticals and other medications come from plants, from nature... I feel that I do believe in those things... they are compatible.”* (Julia)

Young people also addressed the issue of the harm caused by *envy,* which some referred to as *witchcraft*. Regarding this construct, Julia acknowledged the possibility that people could cause harm to others out of envy, saying, “just as there are good thoughts, there are also bad thoughts.” However, she limited this possibility to people who actually believe that they can be affected by the harm directed toward them, and that the person wishing them harm must be someone close to them. She noted:

“*...I say, no matter how much they do* [witchcraft] *to me, nothing is going to happen because they’re not feeding me, they’re not worrying about me, they’re not looking out for me, I’m the one who’s rushing or getting worked up about my own things.*” (Julia)

She also pointed out that this differs from the beliefs of other people in her community, who consider that envy can affect them regardless of the conditions she mentioned.

Although most of the young people did not explicitly discredit traditional medicine, nor did they decisively deny its potential effectiveness, those with university training in health-related fields showed further distance from the use of traditional medicine. They questioned its effectiveness concerning certain conditions and practices (such as psychosis and suicide), favoring the concepts and approaches of scientific medicine, thus reflecting their internalization of a scientistic logic.

“*My partner’s brother has psychosis, something like that, but it’s because of stress* […] *his parents told me ‘he went crazy because they put a spell on him, because they hate us so much’* […] *and they took him [to a witch doctor] and he kept getting worse* […] *I always told them ‘it’s good that they’re doing healing rituals, putting those candles or burying a lock, but we also need to rely on science.’ When they saw that he was becoming aggressive and things were getting out of hand, that’s when they told me ‘yes, let’s take him.’ They need to reach a certain point before they accept science or any other approach that’s not from their world.*” (Julia)

It also seems that, in these cases, they assume an evolutionist mindset, where the acceptance of biomedical categories of mental illnesses is seen as something that will eventually reach their rural communities, which they consider desirable as it would align with a correct approach to reality. One of the young women explicitly linked this way of thinking to her professional training.

“*Like,* [in the communities] *this idea that there are pathologies that affect the mind and that a person could be physically healthy but not mentally… isn’t really there yet* […] *in cases where someone has committed suicide, they didn’t have that concept of a mental disorder, which in this case could be depression. So, the first thing they’d say was ‘he doesn’t want to work’ or ‘he took his life because he didn’t want to support his children’* […] *but, in reality, it was [a mental disorder], but I think my mindset changed since I started studying nursing.*” (Paula)

In cases of long (four years or more) trajectories of depressive disorders, anxiety, and substance abuse), some mentioned that they sought traditional healing during their childhood or adolescence at the decision of their parents, who assumed that their condition could be treated as a result of fright or witchcraft. However, they indicated that it was ineffective in treating their suffering.

As one can see, it is possible to point out that the young people engaged in traditional medical practices, accepted some of its nosological categories, but distanced themselves from how their etiology is conceived in their community. Regarding the effectiveness of these therapeutic resources, in some cases, they explained the possibility of real efficacy, drawing on the foundations of scientific knowledge.

### Westernized forms of care

Living in an urban context facilitated the accessibity to different forms of care. Proficiency in Spanish, the use of information and communication technologies (primarily the Internet), a higher-than-basic education (in most cases, even with a university degree), and access to social security or personal/family resources supported access to paid private consultations. In the urban setting, the interviewees reported having used general medicine services, psychology, psychiatry, and mutual help groups. This section presents some findings, focusing on the cultural appropriateness of the selected therapeutic options. It is important to note that in most of the accounts, barriers to access this type of care or dissatisfaction with previous biomedical treatments did not emerge spontaneously in the narratives as an accessibility barrier or as a reason for dissatisfaction; however, when we raised the topic, the interviewees did share having experienced these situations.

#### Biomedical care

The young people reported seeking a general practitioner or emergency services when they attributed their illness to an organic disorder, or when they clearly experienced physical deterioration due to emotional distress. This form of care did not prove significant for addressing the young people’s distress; however, it helped rule out an organic problem and directed their health-seeking trajectories toward psychology services through either recommendations or formal referrals.

#### Psychological care

In the urban setting, psychological care was available and, from the interviewees’ perspective, accessible in both public and private services. The cost per session in private services ranged from 100 to 500 Mexican pesos (about $7 to $30 USD), which did not represent a significant economic barrier, although in some cases it was considered an excessive expense for their monthly budget. Six of the interviewees sought psychological care at least once during their care trajectories.

Young people accessed a variety of psychological services. Their accounts highlighted religious interventions, strategies for stress management, motivational approaches, and gender and intersectionality-based psychotherapy, which was the most appreciated by the young women interviewed. The psychologists interviewed confirmed that there is a diversity of approaches within their discipline.

Regarding the cultural appropriateness of the services, some professionals in the field were found to define Indigenous identity solely by linguistic criteria or place of origin, while disregarding the range of identity markers that the young people used to shape their ethnicity. One psychologist referred to this identity in the past tense, stating, “*Because Indigenous, the race, the race of Indigenous people, they don’t even exist anymore, we are all mixed*” (conversation with psychologist during observation).

Some professionals also held a stereotypical view of Indigenous people as those who live in rural and marginalized contexts, are poor, and have low educational levels. While it is true that this reflects the reality for many Indigenous people in Mexico, these notions reveal a lack of understanding of the diversity - and inequalities - within this group.

The perception of cultural acceptability regarding this form of care was variable among the interviewees. In some cases, women identified a gap between the recommendations made by the professionals and the possibility of implementing them due to their own cultural context.

“*They treated me like just another person who lived in an urban area* […] *they didn’t take into account that I came from an Indigenous background and that mental health issues are very invisible, and that the fact that my anxiety erupted because I couldn’t express my feelings and emotions could be related to the context in which I grew up* […] *in these types of cultures, it’s very difficult for people to express what they feel, their feelings and emotions freely.* [It’s difficult] *for parents to talk to you about mental health, about trust, about discussing problems* […] *the fact that I can’t express my feelings is something I’ve grown up with, so I think that when they gave me these strategies like ‘talk about it, share it,’ they didn’t have in mind that it would be hard for me.”* (Paula)

However, both the young people and the professionals pointed out that psychology offers a variety of theoretical frameworks, and that contextual, intersectional, or narrative approaches provide tools to address cultural diversity and social inequality. These approaches start from the acceptance that social and phenotypic characteristics lead to different forms of oppression and, therefore, to specific care needs.

“*She* [the psychologist who treats her] *is aware that women are different and that depending on our situation, we have different needs, so I’ve never felt that the theory she talks to me about is just for white women, or that I can’t apply it because of the… the context in which I live, or that I have a requirement like ‘you have to do this and this’ that seems… I mean, that it would be impossible to do* […] *it’s about identifying the types of oppression or discrimination that each person experiences, taking into account their own characteristics. I mean, an Indigenous person doesn’t experience the same type of oppression as a white person, a Black woman doesn’t experience the same type of oppression as a woman… I don’t know, a European woman with money, it’s about identifying our social, physical characteristics, everything that makes you who you are, and understanding that because of that, you suffer from different things.*” (Alma)

It is notable that, in some trajectories, young people sought psychological care more than once, and their experiences varied depending on the approach and the personality of the person providing care. Both women and men mentioned that some of the professionals they saw did not understand them, while some women noted that with some therapists they were able to establish a relationship based on listening and trust. In some cases, particularly among the women, there was a recognition that this form of care is effective and they accepted their diagnosis, although it was also common for them to reinterpret it. In other cases, mainly men, they were unable to trust their therapists and share their lives and problems with them. As a result, psychological care was not effective for them, and they continued seeking relief through other avenues.

#### Psychiatric care

In the Oaxaca Metropolitan Area, psychiatric care is available, but its cost, ranging from 700 to 1,000 Mexican pesos per consultation (approximately $40 to $60 USD), becomes a barrier to effective access for many. Only one of the women interviewed used this type of care, along with one of the leaders of a mutual aid group who sought psychiatric care in her youth. Both resorted to private services, despite the cost. In both cases, they turned to psychiatric care upon the recommendation of another therapist (either a doctor or psychologist). Those who sought psychiatric care agreed that one of the main characteristics of this approach is the pharmacological treatment based on a diagnosis centered on the organic dimension of their condition.

The experiences shared by the interviewees were varied and they provided examples of the inadequacy of this therapeutic approach for their life circumstances. However, these circumstances did not significantly affect their satisfaction or their decision to continue or abandon the treatment.

We also interviewed a psychiatrist who acknowledged the importance of culture in constructing mental illness and making an accurate diagnosis. However, she recognized that her specialty training did not provide her with the tools to address this cross-cultural issue.

“*A woman claimed that there were spirits in her house. Well, in my psychiatric evaluation, I have to rule out whether she is psychotic, but it turns out that the entire family and the community share the belief in spirits, and they even revere them and show certain respect towards them, and that is normal. For someone else, it might seem like she has a psychotic disorder... I believe there is very little knowledge about what adaptations should be made.*” (Psychiatrist, private practice)

This professional, while acknowledging the role of social factors in the construction of psychiatric conditions, argued that only a multidisciplinary clinical practice, where psychology addresses the social aspects, would enable care that integrates the cultural dimensions of the patient.

The young woman who sought psychiatric care had different experiences with two psychiatrists. The first, a male psychiatrist, whom she consulted at her mother’s suggestion, communicated her diagnosis from the very first visit and prescribed medication. Her experience with this psychiatrist was negative, as the diagnosis frightened her, and the medication caused severe side effects.

“*He told me that I had generalized anxiety disorder with panic attacks, and at first, the diagnosis was very scary because I had no idea about any of it, so it made me feel bad as well. Then came the medication, which I think he never properly regulated for me because, when I started taking it, I was very young, I was nineteen, and I felt very out of touch with the world. I didn’t understand when people spoke to me, and after a while, when I spoke, I would say things in a disorganized way, or say things that had nothing to do with the conversation... I had no emotions, I didn’t cry, nor did I want to kill myself, but I didn’t do anything else*.” (Alma)

In response to the adverse reaction to the medication and the psychiatrist’s failure to return her calls, her parents decided she should discontinue the treatment. Years later, she sought help from another psychiatrist, recommended by her treating psychologist. This psychiatrist confirmed the previous diagnosis, added “*obsessive-compulsive disorder episodes*”, and also prescribed pharmaceuticals. However, this experience was positive because the doctor explained the reasons for medicating her, the possible side effects, and closely monitored her response to the medication to adjust the treatment as needed. She also mentioned that the coordinated care between the psychologist and the psychiatrist was yielding good results.

#### Mutual aid groups

Mutual aid groups are gatherings of people who share a similar problem and come together to share their experiences, typically based on a structured program without direct involvement from professionals. In the Oaxaca Metropolitan Area, groups such as Alcoholics Anonymous and Neurotics Anonymous are available, and they can also be found in rural towns. Accessing these groups does not require payment or bureaucratic procedures, which makes them more accessible.

The three young men interviewed found these spaces valuable for managing their illness. They emphasized the understanding, respect, and the opportunity to express themselves freely in a confidential setting. Only one young woman attended a version of this form of support called the “Fourth and Fifth Steps,” which involves a voluntary retreat for several days. However, she described the experience as negative, noting that participants shared their stories in a loud and vulgar manner, the therapeutic activities scared her, and she felt disconnected from the group, as many spoke about experiences that were foreign to her (such as committing crimes or involvement in drug trafficking).

“*It’s like a retreat, they take you to a place where there is nothing, and they blindfold you so that you don’t know where you are. They convince you that they are going to put you in a place where there are aggressive dogs, and you hear the sounds of the dogs at night in the middle of nowhere, and you are really scared. Then when you enter, there are only tables, but before that, you sign a consent form saying that you wanted to do this. There are tables where they sit everyone down, they give you a sheet of paper and a pencil, and then they start talking about different topics. For example, they say, ‘Now we are going to talk about jealousy,’ or maybe abuse. Then, someone who has already been to the retreat and is now one of the speakers comes in and starts talking about their story, but with words… I don’t know, it’s all so lacking empathy, with swear words, with yelling. It’s like people who haven’t addressed their problems and think that doing it this way is going to achieve something, but they are still having crises while talking about it in front of you. They yell and cry… and since it’s mostly people struggling with addiction who go there, they’ve done really strange things.”* (Alma)

Regarding cultural relevance, these mutual help groups attribute a universal character to the mental illnesses they treat. They assume that, although the specific manifestations may vary, the illness itself remains the same, and thus the therapy does not need to be adjusted for particular social groups, such as Indigenous youth. The only adjustment required would be to provide translation in cases where there is a language difference. Similarly, the young people did not identify any cultural discrepancy affecting the effectiveness of the therapy. Only one person mentioned that some of the everyday experiences they shared in the meetings did not resonate with their group peers, as others had no experience with them (for example, when sharing stories related to farming). However, they did not believe this affected the therapy’s effectiveness. Another interviewee thought that in the mutual aid group in his hometown, they did make cultural adjustments, including language use and different ways of thinking. However, they felt that in the group they attended in the city, no cultural adjustments were necessary.

“*…there in the village, because of the group I’m in, I realize that one, well, adapts, because they speak Chatino, so you have to speak Chatino, and understand that their way of thinking is different, or we think differently when we are in the village - well, today I had to go to the hill to talk to God - and here, not… because the culture is different*.” (Fernando)

On the other hand, some highlighted as a positive aspect the similarity between the conception of alcoholism in Alcoholics Anonymous - understood as a physical, mental, and spiritual illness - and the way people in their indigenous communities view individuals as physical, social, and spiritual beings. Another element that seems to favor the cultural acceptability of this therapy is its foundation in sharing life experiences, which aligns with the changing and culturally diverse realities of the various communities where this form of care is implemented.

## DISCUSSION

The findings of this study align with theoretical approaches to youth as a historical construction that refers to both social classification and group identity.^62^ This construction, among the study participants, reflects a Westernized way of representing and experiencing youth, which may conflict with the conceptions of the life cycle prevalent in the rural and semi-urban contexts from which they originate. Additionally, they experienced their ethnic identity in an ambiguous and conflicting manner, closely related to the contradictory attitudes of an urban environment where both the recognition and denial of Indigenous cultures coexist, along with a simultaneous celebration and devaluation of these cultures.

The young people identified as Indigenous, using self-adscription as the criterion, referring to traditions and customs shared with their community, and emphasizing, as observed in previous works,^63^ the importance of belonging to a group defined in ethnic terms, such as family, relatives, or the community. Similar to what another study identified among high school students in Oaxaca,^64^ these young people assumed an ethnic identity based on cultural differences but expressed discomfort with the term “Indigenous,” associating it with experiences of rejection and disdain in urban life.

Previous studies have highlighted the impact of the migratory experience on the mental health of Indigenous people due to contact with an external culture, discrimination, and the marginalization they often experience in cities.^16^^,^^19^^,^^64^^,^^65^^,^^66^^,^^67^ However, this study found that the narratives of the young participants constructed mental health disorders throughout their entire life trajectories, including in their communities of origin. Therefore, we could explore the role of Indigenous cultures in the production of mental health issues.

One way to characterize these cultures is through the category of “communalism,” which refers to a form of social organization based on kinship relations, reciprocity, communal labor practices, and the real or symbolic control of inequality.^68^ We recognize that these aspects can contribute to collective well-being, but we also propose that they can negatively impact individual well-being through dynamics such as envy, which acts as a mechanism of social control.^69^ They can produce a particular type of school bullying or devalue individual freedom in the face of family unity, potentially allowing for impunity in cases of sexual violence within the family. These findings align with previous studies^18^^,^^70^^,^^71^^,^^72^ that have highlighted the negative impact of certain traditional practices - such as forced child marriage or female genital mutilation - and other forms of violence (physical, psychological, economic, and sexual) on the lives of Indigenous girls and women, as well as on their sexual, reproductive, and mental health. We suggest that failing to recognize these shortcomings may constitute a subtle form of racism, as it attributes stereotypes to Indigenous peoples that prevent the acknowledgment of their complexities.

Upon integrating into the city, these young people faced a different sociocultural context and had to make both pragmatic and symbolic adaptations. Classic authors have often seen the city and its way of life as a threat to the mental health of its inhabitants. For Kraepelin, with an organicist approach, the conditions of modern life in large cities led to the degeneration of individuals and races, and one manifestation of this degeneration was precisely mental illnesses.^73^ From a more sociological perspective, Simmel attributed to urban life at the beginning of the 20th century the ability to produce important transformations in the spirit of individuals, analyzing the contrasts between rural and urban life.^74^ More than a century later, the essence of the contrasts identified by Simmel seems to remain relevant in the experiences of the interviewees, who identified oppositions between life in their rural towns and in the urban environment of the Metropolitan Area of Oaxaca. For them, the city was synonymous with freedom, speed, and anonymity, while life in their town was slower and, in terms used by Simmel, characterized -not always positively - by strong social bonds, as well as limitations on individuality. These contrasts posed significant challenges for their adaptation to urban life.

However, it is important to clarify the type of migration experienced by the young people in this study to better understand its impact on mental health. The reasons for migrating were related to their expectations of continuing their studies and improving their quality of life, expectations that were largely fulfilled. These reasons contributed to experience their arrival in the Metropolitan Area of Oaxaca as a desired and valued event, while not all aspects of the culture they left behind were painful to lose. Additionally, we should consider that theirs was a regional migration from the countryside to the city, which facilitated frequent returns to their communities of origin. This physical and symbolic “back and forth” allowed the young people to maintain close ties with their families and communities, easing the experience of grief and uprootedness that typically characterizes international migrations.^75^ Also, their youth, the fact that they were single and without children implied that the family they left behind was their family of origin, not a family they had created, which led to a different experience than that of adult migrants, who often leave behind partners and children and tend to yearn for the family life they left in their communities of origin.^75^ For most of the young people in this study, their imagined future lay outside their community.

These elements can help explain the migratory experience of the participants in this study, which represented a challenge for adaptation, but did not, in itself, constitute a significant source of social suffering. The peculiarities of their arrival, however, were identified as the source of their psychological suffering, which led to what they recognized as the emergence of psychopathological illnesses.

These peculiarities included an ambiguous urban attitude toward their ethnic condition, in conjunction with other forms of discrimination such as colorism and classism, as manifestations of the ideology of white privilege, which, as previous studies have pointed out, contributes to the establishment of racial hierarchies in our country.^75^

This attitude was characterized by a discourse of respect for differences, while simultaneously fostering subtle or not so subtle expressions of violence and discrimination in the various spaces where the study participants lived their lives, particularly in schools and in health care services. It is important to note that this discrimination did not necessarily stem from an outright denial of equal rights, as these young people did have effective access to both public and private educational and healthcare institutions. Rather, it consisted of a set of practices that produced unequal and mixed outcomes, affecting access to certain rights and thereby reproducing social inequality, which, according to Solís,^76^ can also be considered discrimination. This highlights the importance of understanding the attitude of the host culture toward immigrants to comprehend the impact of migration on mental health.^16^. It also reaffirms the need for mental health interventions in these communities to include strategies aimed at dismantling these forms of violence, which, as previously noted, range from interpersonal aggression (what Scheper-Hughes called “everyday violence”) to forms of structural violence related to the political and economic organization of society that imposes conditions of oppression and inequity on individuals.^77^

The disorders resulting from these dynamics were categorized as anxiety and depression, substance abuse, and suicidal thinking, which are generally referred to as “minor” or “non-severe” problems. Their prevalence increased during the COVID-19 pandemic and different containment strategies were developed.^78^ These problems are also the most prevalent for this age group^79^ and among adolescents living in Indigenous communities.^67^

Unlike studies that have identified Indigenous conceptions of the person that deny the separation of mind and body^26^^,^^65^ and have analyzed cultural syndromes with both psychological and organic^26^^,^^29^ manifestations, this study identified a process of “Westernization” in the ways of naming, explaining, and thus producing mental disorders. This may be due to the fact that, as the participants integrated into urban life, they took on specific roles (student, boyfriend, girlfriend, friend) in particular spaces (school, places of leisure and fun), and they were exposed to “youth” concerns that produced emotional distress. They named, elaborated on, and gave meaning to this distress, using nosological categories derived from mainstream education, media, and healthcare ideologies to which they were exposed and which are part of the hegemonic psychological-psychiatric knowledge prevalent in the urban context. This finding aligns with previous studies^80^ on the complex dynamics of de-indianization among Indigenous people who integrate into urban life in Peru. These studies identified that urban Indigenous people maintain a self-consciousness of otherness based on various cultural markers, while they integrate forms of knowledge from the urban context, to which their own knowledge systems are often subordinated.

It has been noted that Indigenous populations face barriers to accessing healthcare services due to their rural residence, as well as their poverty and marginality.^19^^,^^24^ However, this study worked with a group of young people residing in an urban context, and whose social position is not - in most cases - marginal. This facilitated access to various forms of care, both public and private; at the same time, we identified another type of exclusion that affects not the people themselves, but their knowledge.

Previous studies on the healthcare trajectories of Indigenous people with general or mental health issues^42^^,^^43^^,^^44^^,^^51^ reported a combined use of Indigenous medicine and biomedicine throughout the trajectory. However, this study identified a limited use of traditional medicine and a limited understanding of its theoretical and symbolic underpinning, or rather, a significant reinterpretation of this knowledge, which now participants articulated with a scientific and evolutionary logic to explain the production of mental disorders and their treatment. This process seems to run counter to what collective mental health proposes: the recognition that the mental should not be considered separate from the organic,^81^ and a critique and distancing from scientific logic.

Regarding the characteristics of the trajectories, we identified some similarities with previous studies conducted in Argentina, Chile, and Mexico,^46^^,^^47^^,^^48^^,^^49^^,^^50^ despite the fact that these studies worked with different populations (for example, adults, children, or juvenile offenders, none of which were Indigenous) or with different mental health issues (such as developmental problems), and focused only on substance abuse as the primary disorder, or limited themselves to studying institutional trajectories within the official healthcare system.^47^

Among the similarities, the heterogeneity of the analyzed trajectories stands out, both in terms of the duration and the variety of selected forms of care, such as public and private psychological and psychiatric services, professionals (psychologists and social workers) from educational and governmental institutions, and group and holistic therapies. Regarding how participants evaluated the forms of care they used, they shared a criticism of the lack of empathy from some professionals, their impersonal treatment, their limited understanding of the disorder afflicting their clients, the limited usefulness of some of the tools they provided, and the asymmetrical relationships between specialists and patients (especially in the case of psychiatry, which was the most criticized form of care). On the other hand, they positively evaluated horizontal and empathetic professional-patient therapeutic relationships, smooth communication with psychiatrists to overcome mistrust of medication, and collective interventions (even those conducted within hospital institutions) that allowed them to learn about their peers’ experiences and facilitated trust and understanding between peers. They also valued holistic therapies such as meditation, yoga, homeopathy, and acupuncture. These options, though, are generally used by middle-class sectors and were not utilized by the participants in our study; however, they did value positively some elements related to these therapies that they found in mutual aid groups, such as the integration of mind and body and the pursuit of self-knowledge and spiritual transformation.

Our study on Indigenous youth, provides some new insights, notably the central role of violence related to racism and discrimination in the construction of mental disorders; the role of traditional knowledge and ethnomedical resources in addressing such disorders and in self-care in general; as well as specific challenges that Indigenous youth face in accessing their preferred forms of care, due to their specific positionalities at the intersection of race or ethnicity, social class, and in some cases, gender.

We also identified that, despite the increasing presence of Indigenous people in cities and the growing recognition that these groups are socially differentiated internally,^82^ difficulties persist in the institutional recognition of their diversity and complexity. In psychological services, professionals tend to take for granted that language and place of origin define who the Indigenous people are, while they stereotype them as poor, racialized, and anchored to rural communities. This finding is similar to other works that highlight stereotypical and reductionist representations of Indigenous people as peasants and illiterate;^80^ it also echoes parallel difficulties on the part of professionals and policy-makers in defining and labeling who is “Indigenous” in official sources of information,^83^ revealing a systematic pattern of invisibilization that ends up discounting their specific needs.

This inability in addressing diversity characterizes the psychiatric model of care as well as the more individualistic psychological approaches that subscribe to the biologicist and universalist conceptions of mental illness. We propose that this inability is a manifestation of a broader limitation in both current psychiatry and those forementioned psychological approaches in understanding the sociocultural determinants of mental health. This phenomenon echoes what some authors have termed the “neokraepelinian rearmament” in U.S. psychiatry in the 1970s (which, in turn, produced counter-resistance) characterized by a return to the modern positivist view that focuses on biological research, the drive for the classification of mental disorders, the expansion of psychopharmacology, and the medicalization of life in general.^84^

Some authors^19^ interpret this situation in terms of cultural inadequacy, as mental health services don’t integrate in their approaches the worldview and ways of life of Indigenous peoples. However, we argue that this interpretation does not take into account an element that was very much present in the dynamics we studied: the naturalization of the superiority of Western knowledge over non-Western knowledge. We then prefer the conceptual category of epistemic racism^85^which presumes an imposition of knowledge in the field of “mental health” through what is known as the “internal coloniality of psychology”:^86^ a discipline that legitimizes a system of representations aligned with the project of modernity^87^ and at the service of the reproduction of capital.^85^ These representations permeated the imaginaries of the interviewees and help explain why, despite the fact that they referred to situations that evidenced a lack of cultural adequacy, they did not interpret cultural inadequacy as a barrier to care nor did they attach any importance to it. These attitudes on the part of this youth evidence the incorporation and naturalization of the social demands to adapt to the prevailing conceptions, dynamics and values of the urban space where the interviewees live. 

Regional health policy in the Americas gives priority to reversing these discriminatory dynamics and recommends guaranteeing the human rights of people with mental health issues, addressing gender, racism, and discrimination as determinants of mental illness.^88^ From different institutional, political, and academic fronts, the intercultural approach has been proposed as a way to achieve these goals. The region has taken actions in line with the approach, such as the establishment of intercultural hospitals, the development of transcultural psychiatry departments in research and healthcare centers, the inclusion of cultural syndromes in the DSM-5, and proposals for culture-adapted instruments to explore aspects of mental health in Indigenous populations.^24^^,^^89^ Despite the promising nature of these proposals and actions, it will be necessary to address the gaps and contradictions between official discourses and actual practices,^90^ as well as the risk of instrumentalizing the intercultural approach to serve the logic of reproducing existing relations of domination.

## CONCLUSIONS

The Indigenous participants in this study experienced global youth issues, but these were nuanced by their ethnic specificities and the local contexts where they live, where prevails an ambivalent attitude towards Indigenous people and being Indigenous. These conditions led to specific ways of falling ill, naming the pathological condition, and addressing it, marked by the Westernization of their conceptions and experiences and by overcoming barriers to mental health services, which are accessible although with limitations regarding cultural relevance.

These dynamics revealed the transversal action of what we call structural racism, which hierarchizes people and knowledge, and which permeates ways of life, personal interactions, institutional dynamics, and both professional and popular knowledge. Racism can also manifest in conceptions that romanticize communal life and fail to explore its role in producing psychological distress.

We propose that the role of racism in the construction of mental disorders and their care exceeds the generally recognized forms, which focus on personal interactions between peers and between healthcare professionals and patients. While this form of racism persists, is harmful, ad requires concerted efforts to overcome it, it is important to identify other expressions of racism, such as the invisibilization of Indigenous individuals and collectives and the coloniality of knowledge.

It is important to clarify that the critical approach we chose to analyze the processes of deindianization or Westernization of the ways of life and forms of thinking of Indigenous youth, and their relationship with the construction and care of mental disorders, does not imply an essentialist stance that rejects cultural change. What we question are the conditions of that change, characterized by the hierarchization of knowledge and ways of life that render individuals and their needs invisible, and that favor an adaptation to hegemonic knowledge, which imply moving in the opposite direction of what the intercultural approach proposes.

Some of the psychological therapies and forms of self-care mentioned by the study participants show theoretical or procedural promises for the cultural diversity approach and against social inequality. For future research we suggest to assess their potential for developing decolonial approaches in the field of mental health and illness, and to strengthen interdisciplinary dialogues between psychiatry and psychology, on the one hand, and other social sciences, including sociology and medical anthropology, on the other, in order to transcend academic boundaries and to contribute to ongoing training for professionals responsible for mental health care.

Finally, we acknowledge that a limitation of this study was the difficulty we encountered in contacting the study participants which affected and reduced the variability of the sample. We attribute this difficulty to the double stigma surrounding self-adscription to an ethnic identity and the recognition of suffering a mental disorder, limiting visibility within the collectives of individuals with either of these characteristics. Another shortcoming was the limited availability of time for the research, which did not allow to conduct interviews with mental health professionals working in public social security or Ministry of Health institutions because of the time required for the institutional research approval. Also, due to time constraints, we could not explore in depth religious-related care; a form of care that emerged during the interviews. For future research, we recommend to incorporate therapeutic spaces and actors related to religion and spirituality, investigate the implications of gender in shaping care trajectories, and address mental health issues among diverse populations in terms of ethnicity, age, residence, and social class. Lastly, we acknowledge a typical shortcoming of biographical studies: the inability to contrast and complement participants’ narratives with other data sources, in order to achieve a more accurate understanding of how the care trajectories actually developed.

## SUPPLEMENTARY MATERIAL

Systematization of the analyzed care trajectories (see Supplementary material).
